# Measuring Longitudinal Genome-wide Clonal Evolution of Pediatric Acute Lymphoblastic Leukemia at Single-Cell Resolution

**DOI:** 10.1101/2025.03.19.644196

**Published:** 2025-03-19

**Authors:** Yakun Pang, Tamara Prieto, Veronica Gonzalez-Pena, Athena Aragon, Yuntao Xia, Sheng Kao, Sri Rajagopalan, John Zinno, Jean Quentin, Julien Laval, Dennis Yuan, Nathaniel Omans, David Klein, Matthew MacKay, Iwijn De Vlaminck, John Easton, William Evans, Dan A. Landau, Charles Gawad

**Affiliations:** 1 Department of Pediatrics, Stanford University, Stanford, CA, 94305, USA; 2 Division of Hematology and Medical Oncology, Department of Medicine, Weill Cornell Medicine, New York, NY, USA; 3 Sandra and Edward Meyer Cancer Center, Weill Cornell Medicine, New York, NY, USA; 4 New York Genome Center, New York, NY, USA; 5 Meinig School of Biomedical Engineering, Cornell University, Ithaca, NY, 14850, USA; 6 Department of Computational Biology, St. Jude Children’s Research Hospital, Memphis, TN, 38105, USA; 7 Department of Pharmacy and Pharmaceutical Sciences, St. Jude Children’s Research Hospital, Memphis, TN, 38105, USA; 8 Meinig School of Biomedical Engineering, Cornell University, Ithaca, NY, 14850, USA

## Abstract

Over 80% of children with acute lymphoblastic leukemia (pALL) can be cured by treating them with multiple chemotherapeutic agents administered over several years, whereas pALL is incurable with 1–3 medications, suggesting significant variation in drug susceptibility across clonal populations. While bulk sequencing studies indicate that pALL cells contain relatively few genetic variants compared to other cancers, the true extent of genetic diversity at the single-cell level remains unknown. Here, we used three complementary approaches to investigate pALL genetic heterogeneity: error-corrected bulk sequencing, single-cell exome sequencing, and primary template-directed amplification (PTA)-enabled single-cell genome sequencing. We discovered that some ETV6-RUNX1 samples harbor multiple independent ras clones and that individual pALL cells harbor substantially more mutations (mean 3,553 per cell) than detected in bulk samples (mean 965 mutations), with variant signatures suggesting both early and late APOBEC-driven mutagenesis in ETV6-RUNX1 patients. Using PTA-based phylogenetic analysis of over 150 single-cell genomes from four pALL patients, we identified heritable phenotypes associated with specific genetic alterations, including some low-frequency clones that are preferentially selected for during chemotherapy treatment. Our findings reveal previously undetected genetic diversity in pALL and suggest that pre-existing mutations influence treatment response, with implications for future therapeutic strategies. This study provides a high-resolution framework for understanding cancer clonal evolution during treatment, yielding important new insights for developing more effective therapeutic approaches for pALL.

## Introduction

The sequential acquisition of mutations is a hallmark of cancer formation, and the underlying genetic diversity created through that process can drive subsequent clonal selection as patients undergo treatment(Black and McGranahan, 2021). Much of what we know about the magnitude, causes, and consequences of cancer clonal evolution results from sequencing tissue samples(Cortes-Ciriano et al., 2022). These bulk measurements have been critical for cataloging cancer-associated mutations and their mutagenic signatures, which can change as patients undergo treatment(Ma et al., 2018). However, these strategies do not determine the true magnitude of that evolution, which occurs within single cells.

Curative treatment of pediatric acute lymphoblastic leukemia (pALL) requires the deliberate deployment of 10 or more antineoplastic drugs in specific combinations over two to three years(Inaba and Pui, 2021). The requirement of such complex treatment regimens suggests that there are significant differences in drug susceptibilities across clones in each patient, requiring treatment with multiple medications with different mechanisms of action to eradicate all clones and successfully cure patients. However, bulk sequencing studies have concluded that pALL samples are genetically quiet, as they harbor few genetic variants at the tissue level compared to other pediatric and adult cancers(Grobner et al., 2018). Still, at the resolution of bulk sequencing, one can detect the presence of multiple co-existing clones within single leukemic samples(Brady et al., 2022; Gawad et al., 2014). In addition, serial bulk sequencing studies have demonstrated that pALL clonal admixtures change over time in response to chemotherapy and that relapse-specific mutations mark leukemic cells capable of surviving standard treatment regimens(Ma et al., 2015). These findings suggest that there is indeed more genetic diversity within pALL samples than one can detect with bulk sequencing. Thus, while pALL has been shown to have some intra-leukemic genetic diversity, existing data have likely revealed only uncovered “the tip of the iceberg” of this critical axis of cellular heterogeneity.

**Figure 1.**
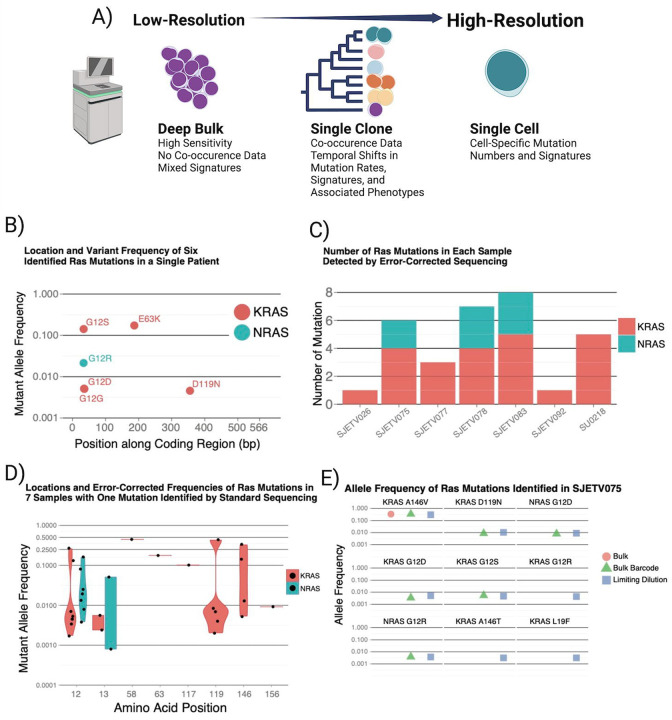
Evidence for large population genetic diversity in *Ras-mutant* pALL. (A) Overview of additional insights we obtained in this study by studying pALL at increasing levels of resolution. (B) Error-corrected sequencing in a patient with 1 ras mutation detected with bulk sequencing identified 4 additional lower-frequency activating mutations. (C) Subclonal *RAS* mutations were also common in a larger cohort in which each patient had one mutation identified in the bulk sample but had a median of 5 activating *RAS* mutations. (C) The allele frequency distributions of *RAS* mutations show no evidence of preferential selection of specific amino acid changes. (D) The increased sensitivity of mutation detection with limiting dilution identifies a total of seven activating ras mutations in a patient that had only one ras mutation detected with bulk sequencin.

For this study, we used three complementary strategies to more deeply interrogate the genetic diversity and evolution of pALL (Figure 1A). First, we used error-corrected bulk sequencing to determine if we could detect mutations not detected by standard bulk methods through deeper sequencing of genomic regions known to harbor mutations in pALL(Schmitt et al., 2012). Second, we performed single-cell exome and whole genome sequencing of primary ALL cells using primary template-directed amplification (PTA) to measure the magnitude of diversity at single-cell resolution and determined how those populations evolved in response to selection pressure created by the introduction of standard anti-leukemic therapy(Gonzalez-Pena et al., 2021). Finally, by creating these single-cell phylogenetic trees annotated with other genomic and phenotypic information, we were able to associate specific clonal populations with treatment resistance(Schiffman et al., 2024). Taken together, this study provides important new insights into the magnitude of true genetic diversity and genome-wide clonal evolution of pALL in patients at single-cell resolution.

## Results

### Targeted Error-Corrected Sequencing Identifies Low-Frequency Driver Mutations in pALL

To more deeply characterize the diversity of known oncogenic mutations in pALL patient samples at diagnosis, we first performed targeted error-corrected sequencing of 50 known pALL mutational hotspots using an index patient that was known to harbor a single high-frequency E63K KRAS mutation (Table S1) (Brady et al., 2022; Gawad et al., 2014). In this patient, we detected a total of four additional lower frequency KRAS and NRAS mutations. We did not identify mutations in any hotspots outside of KRAS and NRAS amino acids 12, 63, and 119, all of which are known sites of activating mutations (Figure 1B, Figure S1), supporting the assertion that these are not random events but are activating mutations in cells that underwent positive selection.

We then expanded our study to a cohort of a total of 13 samples from patients with an ETV6-RUNX1 translocation) (the most comment pALL genetic subtype); leukemia cells from six of which had a single RAS mutation identified by bulk sequencing while the other six did not. We identified a median of five activating RAS mutations in each of the six patients for whom a single RAS mutation was detected by bulk sequencing and did not identify any RAS mutations in the remaining six samples, suggesting that a subset of patients with ETV6-RUNX1 translocations are predisposed to the development of multiple clones driven by activating ras mutations (Figure 1C). Examination of the additional ras variants demonstrated that the mutations also affected known ras mutational hotspots, namely KRAS codons 12, 13, 119, and 146 and NRAS codons 12 and 13, without a clear relationship between mutation location and variant allele frequency, which correlates with the number of cells that harbored the mutation (Figure 1D) (Prior et al., 2020).

The increased number of distinct ras mutations revealed with error-corrected sequencing suggested that even more sensitive measurements would identify additional ras mutations. Rather than sequence hundreds of thousands of cells mixed together, we isolated hundreds of cells from patient SJETV075 followed by error-corrected sequencing, hypothesizing that some ras mutations that may have been just below the detection threshold would randomly be present at slightly higher frequencies in the diluted subsamples (Figure 1E). With this approach, we identified three additional low-frequency KRAS mutations (G12R, A146T, L19F) for a total of nine known activating RAS mutations in patient SJETV075. Our examination of all RAS mutations in this cohort revealed that of the 33 mutations identified, 31 were C-to-T or C-to-G changes. This is in contrast with lung cancer, in which approximately 60% of KRAS mutations are G12C or G12V, resulting from C-to-A substitutions that are seen with tobacco smoke exposure, consistent with a different underlying etiology for pALL (Ferrer et al., 2018). Thus, we found that some ETV6-RUNX1 ALL samples appear to be dependent on ras mutations while others are not. Still, this only evaluates the level of genetic diversity in those two genes at the tissue level.

### Single-Cell Exome Sequencing Identifies Clonal Ras Mutations that Drive Branching Clonal Evolution

**Figure 2.**
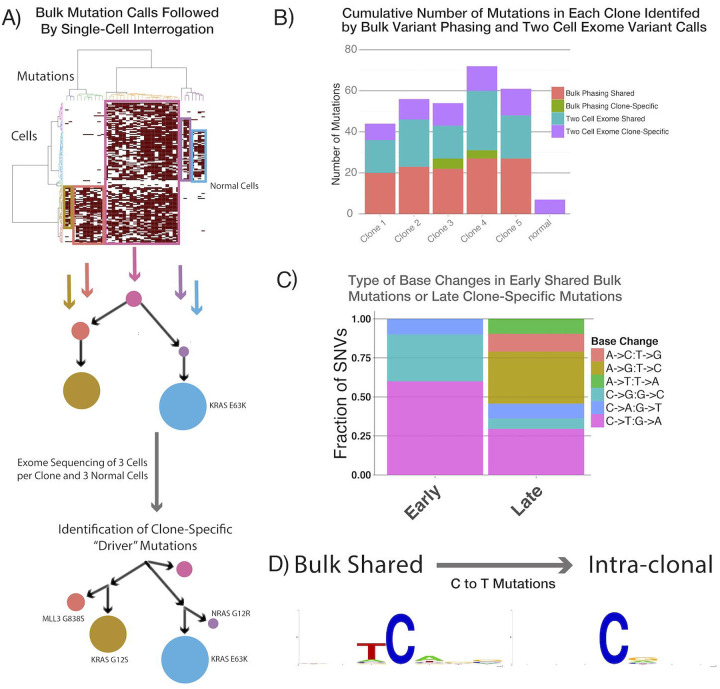
Identification of clone-specific “driver” mutations by using single-cell exome sequencing. (A) The clonal structure of a diagnostic patient sample that was identified by interrogating single cells for mutations first detected in the bulk sample was further resolved by calling mutations in the single cells alone. The clone-specific “driver” *ras* mutations identified as possible causes of the clonal expansions are noted. (B) The number of new mutations identified in each clone using phasing of bulk mutations and 2-cell mutation calls. (C) Base substitution patterns seen in shared (early) and clone-specific (later) mutations. (D) The surrounding motifs in C-to-T mutations in early and late SNVs, showing a the strong APOBEC motif is only present in the early mutations in this patient.

While targeted error-corrected sequencing can uncover low-frequency driver mutations, it does not evaluate the genetic diversity throughout the genome, which is required to accurately determine whether subclones follow nested or branched patterns of evolution. Ras mutations segregating to distinct clones would suggest branched convergent evolution whereby multiple distinct clones separately acquire a fitness-enhancing ras mutation, while a nested evolutionary process where multiple ras mutations occur in the same clone is also a possibility that cannot be formally excluded. To further characterize the genomic diversity of clones, we went back to clones that we had defined in a previous study by segregating variants identified in the bulk samples into single cells(Gawad et al., 2014). To determine if we had missed additional driver mutations that could explain their similar clonal fitness, we performed single-cell exome sequencing on multiple displacement whole genome amplification (MDA) product from three cells from each of those five previously defined clones and on three normal blood cells from the same patient Figure 2A). We achieved a mean saturating coverage breadth of 82% of the target exome with 60 million reads, as compared to 95% coverage of the target exome in bulk samples (Figure S2). Using only the single cells, we called clonal mutations by requiring at least two of the three cells to have the same call at the same genomic position, thus focusing on mutations that enable clonal structure inference.

The initial clonal structure had five high-frequency clones, with one of the two largest clones harboring an E63K KRAS mutation, whereas we could not definitively identify fitness-enhancing alterations in the other clones (Figure 2A). With single-cell exome sequencing, we identified 10 to 29 additional coding mutations per clone with a low background normal cell call rate at a mean of seven coding somatic variants, potentially resulting from clonal mutations acquired in nonmalignant cells, amplification artifacts or sequencing errors (Figure 2B). Importantly, we found a KRAS G12S mutation that was confined to a less abundant clone, as well as another NRAS G12D variant in a separate clone, presumably driving enhanced fitness of those even smaller clones. Thus, we did indeed find that activating ras mutations acquired in separate clones were driving branched convergent clonal evolultion.

We then compared the mutagenic base-change pattern of the earlier, higher-frequency mutations that were detected in bulk and were shared between clones to the later, clone-specific changes that were detected only with single-cell exome sequencing. Interestingly, the early mutations that were shared between clones were strongly enriched for C-to-T and C-to-G changes with an associated APOBEC motif (TpC), whereas the most common later clone-specific mutations were A-to-G and C-to-T without an APOBEC motif (Figures 2C,D)(Mertz et al., 2022). Performing variant calling within single clones identified previously undetectable clone-specific driver mutations, and enabled us to begin to separate out mutagenic signatures over time. However, this approach still had a significant dropout of variant calling capacity as a result of MDA, which is known to create artifacts during the amplification that hamper variant calling, most notably allelic dropout and the distortion of variant allele frequencies(de Bourcy et al., 2014). As a result, we had a relatively small number of high-quality variant calls per cell, limiting the resolution of clonal structures and inference of mutagenic signatures over time.

### pALL Cells have Far Greater Genetic Diversity at Diagnosis than can be Measured with Bulk Sequencing

**Figure 3.**
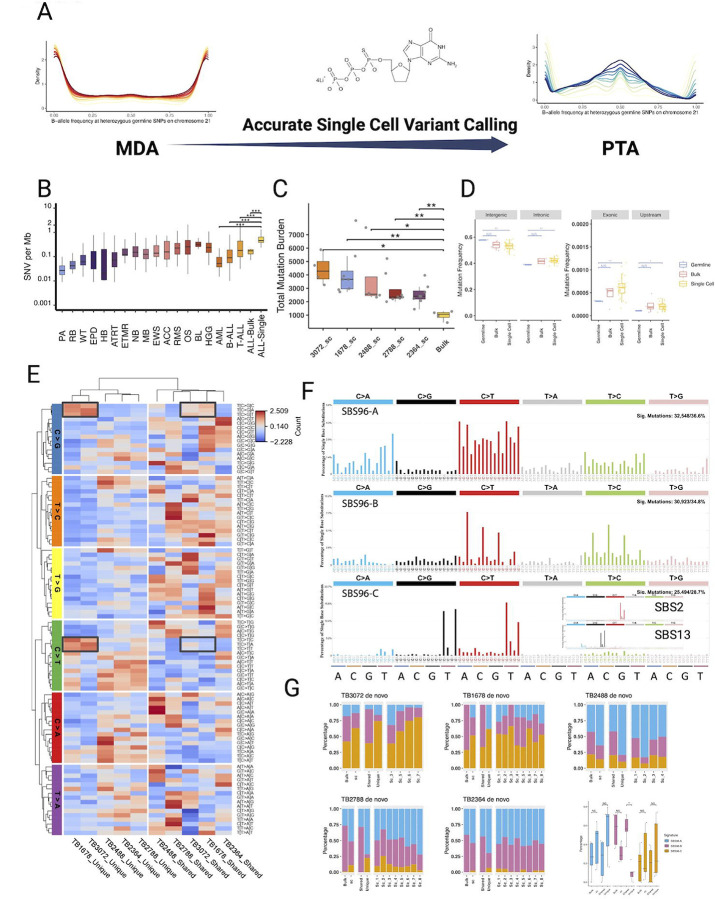
Estimating single-cell mutation rates in ETV6-RUNX1 pALL. (A) The improved allelic balance at heterozygous sites enables more accurate small variant calls. (B) Comparison of a number of called mutations per megabase in a previously published study of pediatric cancers(Grobner et al., 2018) compared to our bulk and single-cell call number from five patients. (C) Close up view of genomewide mutation numbers in five bulk samples and as single cells isolated from the same samples. (D) Comparison of genomic coverage of specific locations to the fraction of bulk and single cell variant calls for each of those locations. (E) Heatmap showing the three base context of unique (late) and shared (early mutations in single cells across five samples. (F) De novo signature detection identified three distinct signatures, with SBS96C showing high similarity to a combination of the APOBEC SBS signatures 3 and 13. (G) The relative abundance of the de novo mutation signatures across patients, which were compared to the relative frequency of germline variants at regions with those annotations.

To improve the quality of single-cell sequencing, we recently developed primary template-directed amplification (PTA), which incorporates irreversible terminators into an isothermal whole genome amplification reaction, resulting in improved single-cell whole genome coverage breadth and uniformity, as well as more accurate variant calling (Figure 3A)(Gonzalez-Pena et al., 2021). To more directly measure the number of mutations in each cell at diagnosis, we performed scWGS using PTA paired with SCAN2, which has been optimized to perform highly specific variant calls in PTA-amplified cells(Luquette et al., 2022). Interestingly, although pALL is considered to harbor few mutations based on bulk sequencing, we found that each pALL cell had a mean of 3553 mutations per cell compared to a mean of 965 mutations detected in the same bulk samples. We then compared those single-cell measurements to the number of mutations estimated to be present in all the major pediatric cancer subtypes revealing that each pALL cell had more mutations than could be detected in bulk samples across all major pediatric tumor types (Figure 3B)(Grobner et al., 2018). We further evaluated the number of mutations in each cell across samples, where we found a range of 2544 to 4424 mutations per pALL cell (Figures 3C, S3). Thus, using accurate single-cell whole genome sequencing (scWGS), we found that each of the tens to hundreds of billions of pALL cells present at diagnosis harbor more mutations than have been measured by bulk sequencing of the same samples.

We then examined the locations of the single-cell somatic mutations compared to germline variants in the bulk samples, where we found an increased density of somatic variants in genomic regions classified as intronic, exonic, and upstream when compared to the density of germline variants in those same regions (Figures 3D, S3B). To further evaluate patterns of SNVs, we created a heatmap of the three base contexts at the SNV locations, which, when clustered, identified a subset of samples from two patients that contained the signature APOBEC C-to-T and C-to-G base changes at TpC sites. In those two patients, the changes made up a larger proportion of the unique mutations, implying that the APOBEC mutagenesis occurred late in disease progression in those patients.

We then performed a more quantitative strategy to confirm this finding by creating three *de novo* SBS96 signatures using Sigprofiler(Alexandrov et al., 2020). We identified SBS96A, which is similar to COSMIC Signature SBS5, as well as SBS96B, which has an enrichment of C-to-T changes at CpG sites. Importantly, isothermal amplification artifacts are known to contain C-to-T changes that are de-enriched at CpG sites, suggesting that these changes are true variants, as reported by others (Lodato et al., 2018; Petljak et al., 2019). Finally, SBS96C has a similarity to a combination of the COSMIC APOBEC signatures SBS2 and SBS13. These findings were confirmed by performing deconvolution of the signatures using the known SBS signatures (Figure S4C–G).

We then examined the three *de novo* signatures across all the samples and cells, where the only significant difference was a de-enrichment for SBS96B in the later unique mutations. Thus, we found that pALL cells have a much higher mutation burden than can be quantified with bulk sequencing and that the mutational signatures can vary at different rates over time in different patients.

### Performing High-Resolution Phylogenetic Analyses with scWGS

**Figure 4.**
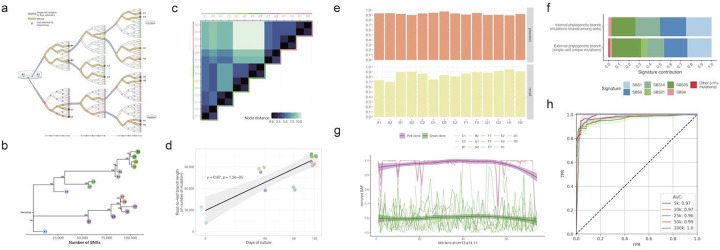
Benchmarking the performance of PTA for scWGS with an in vitro evolution experiment (A) Schematic of the in vitro evolutionary experiment. Four rounds of divisions are represented for the expansion of each single-cell clone (B) Phylogeny of 16 PTA-amplified cells sampled across 7 clones from the evolutionary experiment. Bootstrap values are shown. (C) Node distance of the cells in the reconstructed phylogeny. (D) Correlation between the days of culture of the cells sampled and the branch lenghts of the tree in number of mutations. (E) Recall and precision of the somatic variant calling for the invitro cells. (F) Signature of the mutations mapped to the internal and external phylogenetic branches (G) Mirrored BAF of the in vitro cells (colored as in b) averaged for the heterozygous SNPs contained within 5kb windows which were considered as high-quality for 5 or more cells at a band of the big arm of chromosome 13. (H) ROC curves of the CNV method detection accuracy based on BAF. Cells from the pink clone in b carry a deletion at chr13q14.11 which results in an allelic imbalance (g). Using different BAF fixed thresholds, CNVs were called for every interval accross all cells. CNV calls classified as imbalanced in the pink clone cells are considered FNs and those in the green clone are considered FPs. Solids lines show the mean TPR/FPR for 100 groups of cells selected at random with replacement. Lower and upper bounds of the shady areas represent the mean±2*sd of this value.

We next leveraged our improved variant calling of PTA for high-resolution phylogenetic reconstruction with larger numbers of cells from each patient. To establish technical feasibility, we first tested the ability of scWGS with PTA to provide accurate phylogenetic reconstruction using an *in vitro* evolutionary model (Figure 4A). We deeply sequenced two single cells from a parental DLD-1 cell clone and two cells from seven related clones. Coverage dispersion of the sixteen cells was comparable to the values obtained in the original PTA publication and to the value of single cell colonies established from the same cell line(Poti et al., 2018), as well as lower than MDA-amplified single cells (Figure S5A). Cell coverage breadths for PTA were also comparable to the single-cell colonies (89.4 vs 88.6±1.4 % mean±sd genome) at the final sequencing depths (29.7X vs 34.4X). The PTA libraries showed similar breadth with higher sequencing effort before reaching saturation, which indicates higher library complexity (Figure S5B). The B-allele frequency (BAF) densities for representative PTA (H1) and MDA cell (C6) had distinct patterns, with MDA showing a strong imbalance in the amplification of both maternal and paternal alleles, which was not significantly improved with deeper sequencing (Figure S5C–D). This translated into an expected lower allelic dropout rate for PTA of 5.7±4.2 % compared to 16.4±10.1 % with MDA (Figure S5E), as well as higher germline variant detection recall (Figure S5F). We also compared the number of false heterozygous variant calls on chromosome 21 in this pseudodiploid cell line that should have all homozygous calls to provide an estimate of the false positive rate. The percentage of sites with alternative alleles in the DLD1 H1 cell that likely represent false positive calls is only present at low BAFs, slightly lower than in the MDA cells and similar to the single-cell colony values (Figure S5G).

With our variant calling approach, we detected more than 250,000 somatic single-nucleotide variants (SNVs) across the sixteen cells (mean 15,600 per cell) and recovered a phylogeny that is concordant with the tree structure that had been expected based on the experimental design (Figures 4A–B, S6C). All cell relationships were supported by the maximum bootstrap values shown on the phylogenetic branches. We also found that the two cells from each single-cell clone showed the lowest distance between nodes and that descendants derived from each of the two cells first isolated (two main lineages, pink vs. green cells) also showed a lower node distance (Figure 4C), further confirming our findings. Moreover, we found that the tree branch lengths were correlated to the culture time of the cells in the experiment (⍴=0.85, p-value=3×10^−5^) (Figure 4D). Despite not having designed the experiment to obtain a strictly similar cell generation number at the sampling time for the different clones, we still observed a general mutation acquisition rate that was proportional to time. By applying a linked-read analysis based on the assumption that reads spanning both a heterozygous germline single-nucleotide polymorphism (hetSNP) and a somatic SNV (sSNV) should support only two haplotypes, we estimated our somatic variant calling precision to be 92.4±2.2 %(Bohrson et al., 2019). We also counted the percentage of sites without variant alleles at heterozygous germline sites after having applied a similar filtering strategy as for the somatic calling, and we estimated a recall of 84.4±7.5 % (Figure 4E). Although these results are not directly comparable with previous single-cell precision and recall estimates (they were measured on different cell lines and distinct experimental designs), we added the reported ranges to serve as an indication of the state-of-the-art challenges (Figure S6B).

The use of the phylogeny to discard amplification errors can potentially explain the high precision values we obtained, as has been shown in previous work (Kuipers et al., 2022). Mutations shared among two or more cells (expected to include almost no scWGA errors given that would have to arise independently in different scWGA reactions) and mutations present in just one cell showed similar mutational signatures, further supporting our assertion of high mutation calling precision (Figure 4F). In addition, the signatures with the highest representation included SBS1 and four of the seven mutational signatures that have been previously associated with microsatellite instability (SBS6, SBS14, SBS21, and SBS26). These are most likely true positive calls, given that DLD-1 is an MSI cell line. SBS4 and other rare signatures collectively account for less than 5% of the called variants, potentially because of false positive calls. The fact that the fraction of unexpected signatures is lower than 5% further supports our high precision estimates. Due to the low allelic dropout provided by PTA (Figure S5E), we also developed the ability to detect copy-number variants (CNVs) using the B-allele frequency (BAF) of heterozygous germline SNPs with high accuracy, even for genomic windows as small as 5kb (AUC=0.97) (Figure 4G–H). As an example, the average mirrored BAFs of the hetSNPs present along more than 60 5-kb windows within chromosome 13 band q14.11, revealed that the pink cells lacked one of the two single-cell haplotypes (Figure 4G). Given the improved ability for PTA paired with scWGS to determine mutation numbers and phylogenies in cancer samples, we next sought to calculate the number of mutations in pALL cells using our new strategies.

### Some Phenotypes are Heritable in pALL Cells

**Figure 5.**
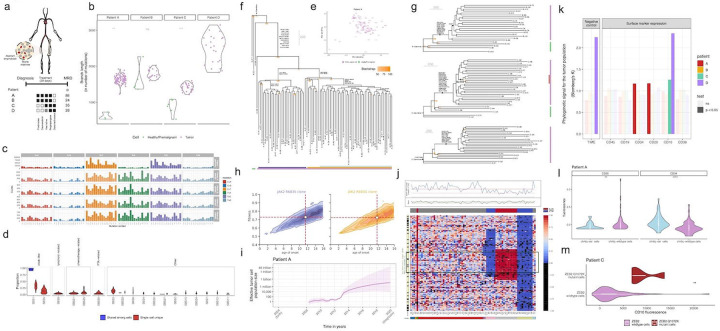
PTA-based scWGS of pALL cells (A) Schematic of BALL patient, number of cells amplified, and treatment each patient received. (B) Estimated number of mutations in normal/premalignant and leukemia cells across all four patients (C) Mutational patterns of DLD-1 colorectal cancer compared to 4 pALL patients. (D) Signatures of shared and unique somatic mutations across patients (E) PCA plots of six phenotypic markers across all cells for patient A showing separation of normal/premalignant and leukemia cells (F) Phylogeny of patient A, showing bootstraps of the branches supported in more than 50% of replicates. (G) Phylogenies as annotations for patients B, C, and D. (H) Posterior distribution of the age of onset of the different JAK2 clones using approximate Bayesian computation. (I) Cell population dynamics of tumor cells along lifespan for patients A, B, and C. Solid line represents the posterior median estimate and the shaded region represents the 95% confidence intervals. (J) Mirrored loss-of-heterozygosity (LOH) events in a 25kb window sorted by clade. (K) Phylogenetic signal of the sorting time (negative control) and surface expression for B-cell lineage markers. Non-significant values are shown in lighter colors. (L) Density plots of CD34 and CD20 fluorescence in a clade carrying a 6q deletion compared to the all other cells. (M) Violin plots of CD10 fluorescence in cells from patient C with or without a ZEB2 mutations.

After validating the ability of scWGS to deliver high-accuracy phylogenies, we chose to focuse on pALL patients that had a suboptimal response therapy where we sequenced more than 150 genomes from pALL cells that had survived induction therapy (Figure 5A). Of note, pediatric pALL is a cancer type with one of the lowest point mutation rates (0.1–1 mutations per megabase by bulk sequencing) and is frequently diploid (Alexandrov et al., 2013), emphasizing the need for high accuracy and coverage scWGS. First, we used the exome data (~60X per cell, ~100X per bulk controls\) to confidently detect somatic mutations in the coding regions of the genome. All patients carried at least one non-synonymous mutation previously identified as driver events in large pALL cohorts in the genes *JAK2, USH2A, MUC16, NRAS, ZEB2, KRAS,* and *FCGBP(Brady et al., 2022)*. Based on the absence/presence of driver exonic SNVs, we further classified cells into premalignant or healthy vs. malignant. In patient D, we did not observe any premalignant/healthy cells; all sequenced cells carried at least a driver SNV in *FCGBP*. We validated the premalignant vs malignant status of the cells by identifying copy-number aberrations. In addition, we performed a PCA of the fluorescence intensity of surface markers per cell (and revealed that the premalignant cells clustered together in patients B and C (**Figure 56E, F**).

We then proceeded with the analysis of the whole-genome sequencing (~8x per cell) and detected almost 130,000 somatic mutations across all patients, observing one or two orders of magnitude more mutations per megabase per patient (10–20 mutations per megabase) than previously reported by bulk sequencing. Our results further highlight the power of single-cell sequencing to reveal the vast subclonal genetic diversity using orthogonal computational strategies. The average number of mutations identified in malignant cells varied across patients (patient A:1497±341, patient B:1049±62, patient C:1180±244 and patient D:2253±333 SNVs per cell) but far exceeded (2.7, 1.3 and 1.6 fold higher, respectively for patients A, B and C) the number of mutations normal B-cells from the same individuals that did not harbor somatic variants seen in the leukemia cells (Figure 5B). The higher mutational load in pALL cells compared to non-malignant cells also suggests that the tumor cells have undergone more cell divisions prior to treatment(Lodato et al., 2015).

The mutational context of the somatic mutations was similar across the four patients (Figure 5C) and clearly different from their germline contexts. With the aim of discovering potential mechanisms behind the patient’s somatic mutations, we performed a signature fitting against cosmic signatures. In contrast to the pALL samples that harbor ETV6-RUNX1 translocations, we found that the vast majority of mutations in these poor responding patients were associated with SBS1. However, many mutations unique to single cells were related to SBS5, another clock-like signature that has been observed in several blood cell studies and may be related to the number of cell divisions (Brady et al., 2022; Kuipers et al., 2022; Machado et al., 2022) (Figure 5D). We also identified SBS7 and SBS9 signatures, previously linked to B lymphocytes(Machado et al., 2022). Interestingly, we found that some of the mutations were consistent with chemotherapy signatures in both the premalignant and tumor cells where we identified SBS21 and SBS25 in variants that were unique to each cell, suggesting they had recently occurred (Pich et al., 2019). Thus, with our single-cell measurements, we can detect differences in the genomes of cells that may be related to their four weeks of induction therapy. This is in comparison to bulk sequencing strategies where you would need to wait for that new variant to expand into a clone that made up at least a few percentage of the bulk sample to be detected which typically occurs months to years later when a patient relapses.

In those patients in which we identified premalignant/healthy cells, we observed that the tumor was derived from a single ancestor cell, i.e. the tumor has a monophyletic origin, as revealed by the exclusive presence of tumor cells in the clades carrying the driver mutations (Figure 5E,F,G). With the aim of learning about tumor population dynamics, we time-calibrated the phylogenies by assuming that each premalignant B-cell descends from a different hematopoietic stem cell (HSC), and under the premise that HSCs are produced from hematopoietic precursors during embryo development (Ottersbach, 2019), we transformed the tree scale from the number of mutations to years using a Bayesian model assuming that branch lengths are Poisson distributed. We then applied a Bayesian non-parametric phylodynamic analysis to reconstruct the cell population size fluctuations along tumor progression for each patient. In patient A, we observed that the tumor population started expanding several years prior to diagnosis, drastically increasing from a few thousand cells up to billions of cells right before diagnosis. However, there is a wide uncertainty in the population size estimates in the years prior to therapy, likely related to extrapolating parameters from less than 100 cells sampled from a tumor composed of tens to hundreds of billions of cells (Figure S6G).

Interestingly, driver events did not only affect the common cancer ancestor but also marked large subclones. For example, in patient A, two major tumor clones harbored different mutations in the *JAK2* gene affecting the same amino acid (JAK2^R683G^ and JAK2^R683S^). Leveraging the high-resolution phylogenetic tree, we calculated the growth rate of the *JAK2* mutated clones using a Wright-Fisher model with selection in an Approximate Bayesian Computation (ABC) framework and consistently observed similar growth rates (Figure 5H). The presence of both clones before and after treatment can be explained by the fact that they both emerged around the same time interval, as was suggested by the ABC analysis. The similarity in clonal timings and growth rates for both clones was further confirmed by coalescent theory. We also detected recurrent copy-number at similar genomic locations that had distinct breakpoints, suggesting that they arose independently, but that the deletion of that region gave those clones a distinct fitness advantage (Figure 5J).

To integrate cell phenotype into our phylogenetic analysis, we annotated each cell in the patient A phylogeny with the expression levels of six different surface markers recorded during cell isolation by flow cytometry. Of note, some of the markers are commonly associated with less mature (CD34, CD10 and CD38) and more mature (CD20 and CD45) healthy B cell differentiation states(Welner et al., 2008). We then used a phylogenetic auto-correlation statistic (Blomberg’s K) to measure if cells more closely related in ancestry would also show a more similar expression of the maturation markers. To explore this question, we assessed which markers showed significant phylogenetic signals in the four patients (Figure 5K). Then, we visually inspected the trees annotated with the significant phenotypes by searching for evident enrichments/depletions of protein expression across well-supported clades. We identified that one clone in patient A exhibited higher CD34 expression and reduced expression of CD20 (Figure 5L). The fact that the expression of the immature CD34 marker was increased while the mature CD20 was reduced, suggested that this clade could potentially carry some genetic factors leading to a less differentiated state. Interestingly, this clade harbored chromosome 6q16-q22 and 6p22.1 deletions, regions encoding for important B cell differentiation factors, including PRDM1, FOXO3 and HDAC2 (on 6q), and a histone gene cluster (on 6p). The loss of the region encoding for histones has been shown to result in a more relaxed chromatin state, which has also been linked to increased expression of stem cell markers (Yusufova et al., 2021). In patient C, we found that cells within a ZEB2-mutant clade show higher expression of CD10, an early B-cell differentiation marker (Figure 5M). ZEB2 has been recently been shown to be required for the formation of age-associated B-cells that have aberrant JAK-signaling, resulting in autoimmunity and other dysfunction (Dai et al., 2024). In patient D, we found that higher expression of CD10 occurred in KRAS^G13D^ cells, a correlation that could not be made without paired single-cell genome and surface marker data. Thus, our data reveals heritable changes in cell differentiation states that may be associated with distinct subclonal genotypes.

### Some Low-Frequency Clones are Selected for after Exposure to Chemotherapy ex vivo

**Figure 6.**
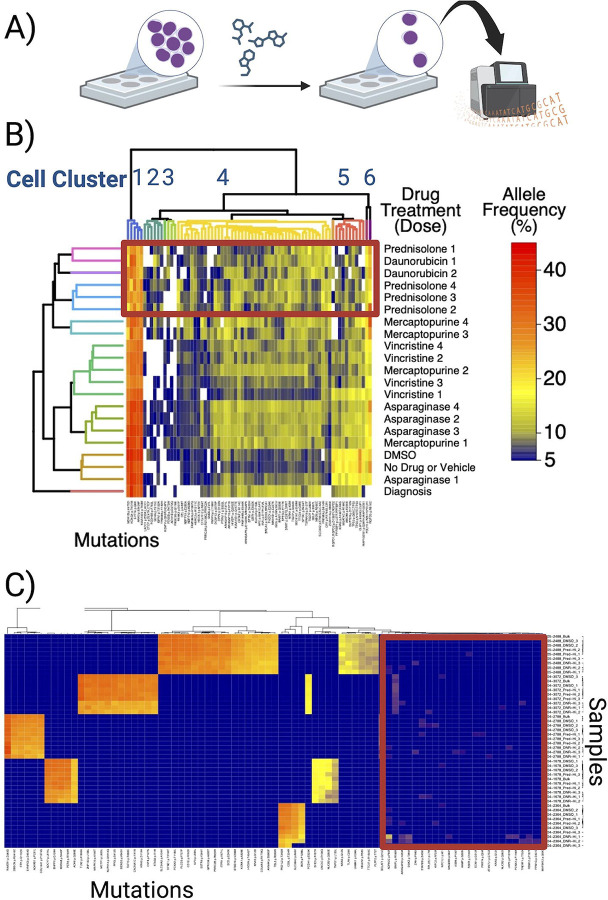
Differential ex vivo sensitivity of leukemic populations to chemotherapy. (A) Overview experimental plan where we treated primary pALL cells with specific drugs followed by bulk sequencing to identify new variants associated with specific drug treatments. (B) Clusters of mutations showing patterns of response to drug treatment and dosage (increasing dose with higher numbers). Cells with mutations in cluster 6 expanded in culture when compared to the bulk sample, while clones with cluster 5 mutations, which includes *KRAS* A146V and two *TP53* mutations, expanded without treatment or upon exposure to low-dose asparaginase. Mutations in clusters 1 and 4 decreased with increasing doses of vincristine, daunrubicin, prednisolone, and mercaptopurine while clones with mutations in clusters 2 and 3 increased in frequency. All cell died at the highest doses of daunorubicin. (C) Explansion of the concept to five additional patients where low-frequency mutations can be detected after exposure to prednisolone and daunorubicin.

With such extensive cellular genetic diversity, we hypothesized that some clones already harbored mutations at diagnosis that altered their susceptibility to treatment. To begin to test that hypothesis, we exposed leukemic cells from diagnosis to 5 standard pALL drugs (mercaptopurine, vincristine, prednisolone, daunorubicin, asparaginase) at four different concentration for each drug using established protocols and used exome sequencing to evaluate the mutational composition of one sample from each drug exposure (Figure 6A)(Lee et al., 2023; Rowland et al., 2023). From this initial exome sequencing screen, we identified 537 putative mutations in at least one treatment. We then performed error-corrected sequencing of those 537 loci with an estimated sensitivity of 0.5% variant allele frequency in triplicate for each treatment to confirm the frequency of the mutations in each treatment. We detected 224 recurrent mutations across the *ex vivo* drug-treated samples (Table S2)—approximately 5 times as many mutations as the 41 that we identified in the initial bulk sequencing.

We then examined patterns of confirmed mutations at increasing concentrations of each drug and performed hierarchical clustering of both samples and treatments (Figure 6B). In general, our hierarchical clustering grouped cells based on the drug received, as well as the dose level. For example, the control cells treated with no drug or DMSO, as well as the cells treated with the lowest dose of asparaginase, showed an increase in a distinct cluster of mutations. This cluster included the highest frequency A146V *KRAS* mutation, along with 2 *TP53* mutations. In a second cluster, treatment with low-dose mercaptopurine or increasing doses of asparaginase resulted in reduced expansion of that clone, while increasing the dose of asparaginase beyond a certain level had no additional effect. This is consistent with the known mechanism of asparaginase, whereby increasing the dose above a concentration that has already removed all of the asparagine has no additional effect on cell killing(Tong et al., 2014). In a third group of clusters contained the increasing doses of mercaptopurine, as well as all dose levels of vincristine. In a final group, exposure to any dose of prednisolone or daunorubicin, as well as the highest doses of mercaptopurine, resulted in a decrease in mutations in clusters 1 and 7, whereas mutations in clusters 2 through 4 increased in frequency. Taken together, these data support the assertion that the underlying genetic diversity of pALL at diagnosis can result in clone-specific differences in treatment response..

To confirm our approach, we performed the same approach on five additional pALL patients using only the highest doses of prednisolone and daunorubicin in triplicate. As expected, each patient sample clustered together. (Figure 6C). In addition, in 4 of five cases, the bulk samples clustered most closely with the DMSO vehicle control and the treatment replicates from each drug clustered together. Taken together, these findings suggest that the samples were clustering based on true genetic differences. We then found distinct sets of mutations that were consistently detected at low levels across treatment replicates, which could be associated with treatment resistance. Still, it is difficult to differentiate these low frequency variants from background technical noise, and we do not have the capacity to determine if any two mutations are in the same cells, as well as the type or state of the cell that harbor those mutations.

### Undetectable pALL clones can be selected for during treatment in patients

**Figure 7.**
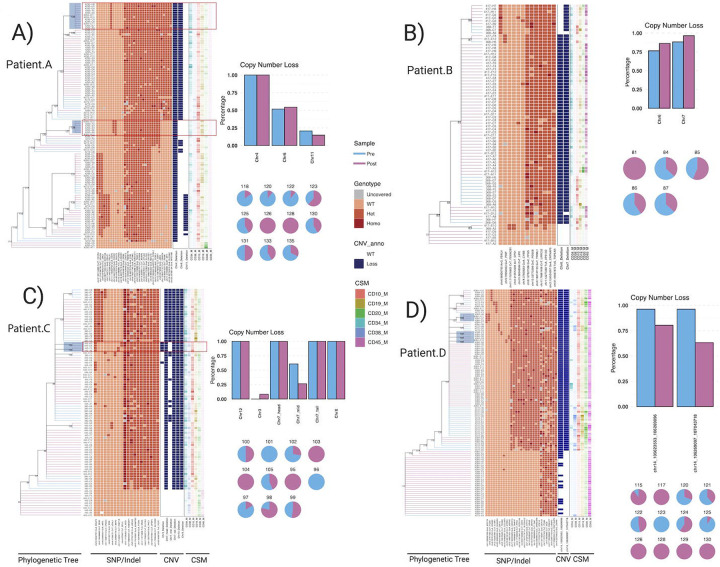
Single-cell sequencing detects clones that undergo selection as patients undergo treatment. Panel A through D depict the phylogenetic trees and associated CNV and surface marker expression. Clones that were not detected in pretreatment are shaded in blue. Relative changes in each recurrent CNV and clonal branch are also depicted for each patient, where it appears that specific SNV are driving the selection of specific clones in patients 4295 and 4084, while CNV appears to be contributing to a small selected clone in patient 445. In contrast to those patients, emergent cloes were not identified in patient 417.

To go beyond listing mutations that increased in bulk pALL samples exposed to chemotherapy while applying our now tools for building tumor phylogenies, we performed single-cell low-pass whole genome for CNV calling paired with exome sequencing while recording the expression of six surface markers, all from the same cells, using the four patients that we previously built post-treatment phylogenies on using scWGS. We sequenced cells isolated from the same patients before and after four weeks of combination chemotherapy with prednisone, vincristine, and asparaginase, with high-risk patients also receiving daunorubicin. With this approach, we were again able to identify premalignant cells, as well as segregate cells into phylogenies based on somatic mutations, and we found that the CNV segregation was concordant with the SNVs and Indels used to build the trees (Figure 7). When we then examined the percent of cells with each CNV before and after treatment, we found that most patients had stable CNV proportions, with the exception of one clone in Patient C (Figure 7).

We then sought to identify specific clones that underwent positive selection as a result in treatment where it is estimated that the leukemic cell burden is decreased from over an estimated100 billion to 10 to 100 million cells(Bartram et al., 2020). With the high level of population genetic diversity we have detected before treatment, we were not surprised to identify SNVs in clones that were not detected in the diagnostic samples. More specifically, across all patients, clones that were not detected in the pretreatment samples harbored mutations in CDHR1, FAM71A, ITGA9, KIF21B, MYL5, NTRK3, NXPH2, SULF2, TENM2, and USP2. In addition, patient C only had one emergent clone during treatment that harbored a previously undetectable 90 Mbp deletion on chromosome 3q that included deletions in a large number of genes, suggesting that both SNV and CNV can contribute to disease persistence during treatment. Importantly, several of these alterations have been previously associated with treatment-resistant ALL. USP2 fusions occur in infant and mixed phenotypic ALL, two of the most difficult-to-treat subtypes of ALL(Meyer et al., 2019). Similarly, NTRK3 fusions have also been associated with a high-risk ALL(Roberts et al., 2018), and ITGA9 expression has been associated with high ALL MRD levels(Shah Scharff et al., 2020).

To further evaluate the potential relevance of our MRD-specific variants in this cohort of four patients, we compared our results to those that studied bulk paired diagnosis, remission, and relapse samples from 92 patients with pALL. Of the ten MRD-specific genes with variants we identified in these patients, we identified mutations in CDHR1 in 4, SULF2 in 4, and KIF21B in 3, as well as NTRK3, ITGA9, and NXPH2 mutations in a single relapsed patient in the previous study. Further, within those genes, nonsilent coding variants occurred in CDHR1, ITGA9, KIF21B, NTRK3, and SULF2 (Table S3). In addition, mutations in ITGA9 and NTRK3 were only relapse-specific (i.e. not detected at diagnosis), as was seen in the six NT5C2 and three PRPS1 mutations identified in the 92 relapsed patients that are known to be acquired to provide resistance to thiopurines(Meyer et al., 2013). Conversely, CDHR1, KIF21B, and SULF2 had variants that could be found at diagnosis and persist to relapse in addition to the relapse-specific variants. This is a similar pattern to other genes in the relapsed cohort with well-established connections to relapsed pALL, such as CREBBP, FPGS, KRAS, NRAS, and NR3C1. In addition, we found that the deletion on chromosome 3q only seen in the post-treatment samples included two recurrent focal deletions found in relapsed patients that contained either BTLA (6/92 patients) or TBL1XR1/LINC00578 (5/92 patients)(Waanders et al., 2020). Taken together, these findings support the assertion that we are identifying mutations in drug-resistant clones after just one month of therapy that may not otherwise be detected until months or years later when a patient relapses.

## Discussion

With this study, we have created genomic portraits of pALL at a higher level of resolution than previously reported, enabling views of the clonal diversity and evolution of the disease not previously available. By driving down the sensitivity of detection with deep error-corrected sequencing, we detected additional oncogenic ras mutations not found with standard bulk sequencing. With single-cell exome sequencing, we built phylogenies that determined that the ras mutations segregated to distinct clones and began to build cellular phylogenies that order mutation acquisition, as well as the timing of mutational signatures. With more accurate single-cell whole genome sequencing using PTA paired with our new analytical strategies for calling SNV and CNV to build even more comprehensive and accurate phylogenies, we were able to provide accurate estimates of the actual number of variants in each pALL cell, which we estimate to be several fold more mutations per cell than reports based on bulk sequencing, which is multiplied across the billions of cells at diagnosis to determine the true population genetic diversity. Importantly, we then show that within that genetic diversity, a subset of clones preferentially survive *ex vivo* exposure to standard chemotherapy, as well as treatment in patients.

Using these technical advancements that provide higher resolution longitudinal view of pALL evolution from diagnosis to the end of induction therapy, we were able to identify MRD-clone-specific variants in CDHR1, FAM71A, ITGA9, KIF21B, MYL5, NTRK3, NXPH2, SULF2, TENM2, and USP2. Importantly, five of those genes, CDHR1, ITGA9, KIF21B, NTRK3, and SULF2, were found to harbor relapse-specific nonsilent coding variants in at least one patient in a cohort of 92 relapsed pALL patients, suggesting we have identified variants that are associated with early treatment resistance, as well as the long-term persistence of clones that ultimately contribute to relapse. In addition, the MRD-specific variants followed two patterns see in relapsed pALL patients. Only nonsilent coding variants in ITGA9 and NTRK3 were seen at relapse, suggesting they only provided a fitness advantage after exposure to standard therapy. The second group of variants in CDRH1, KIF21B, and SULF2 were seen in both diagnostic and relapse samples, suggesting they can provide a fitness advantage in the development of pALL, as well as contribute to the persistence through treatment to cause relapse. Finally, we found a clone that harbored a distinct CNV underwent positive selection during treatment, and that region included BTLA or TBL1XR1/LINC00578, which have both been associated with focal relapse-specific deletions. Importantly, the identification of these variants that have been associated with relapse months or years later after only four weeks of treatment could provide a new path for identifying and targeting those specific MRD cells before they have the opportunity to further evolve resistance to the drugs they will receive or the following two to three years.

Our approach has a couple of important limitations. First, due to sequencing costs, the number of cells per sample was limited. We do not know how many cells would need to be sequenced to meaningfully saturate our genomic interrogation of clones with specific phenotypes, such as treatment resistance. Similarly, we have only evaluated a limited number of samples. Larger cohorts will be required to identify generalizable genomic patterns of clones that are associated with treatment resistance in pALL patients. Our study establishes the rationale for expanding the number of patients and cells studied with these more sensitive methods. In addition, our phenotypic annotations were limited to surface marker expression or survival after exposure to chemotherapy. PTA has recently been combined with whole transcriptome sequencing from the same cells(Chung et al., 2024), which could be evaluated with our recently developed tool PATH, to map entire transcriptomes onto phylogenetic trees to identify changes in the expression of specific genes that are heritable(Schiffman et al., 2024).

In summary, we have created both wet and dry lab tools to enable high-resolution interrogations of cancer clonal evolution as patients undergo treatment that has uncovered far greater genetic diversity than can be detected with standard bulk sequencing. We anticipate that the application of these tools to larger datasets will enable us to begin to create accurate quantitative measurements and models of a patient’s clonal evolution over time, improving our ability to predict treatment response while identifying specific clonal genotypes and phenotypes that can be more efficiently eradicated with new therapeutic strategies.

## Online Methods

### Error-corrected sequencing

Adapters with unique identifiers were prepared as previously described. Aliquots of 250 or 500 ng of genomic DNA then underwent 30 min of chemical fragmentation and standard library preparation by using the KAPA HyperPlus Kit (Kapa Biosystems) with adapters that contained unique molecular identifiers as described(Kennedy et al., 2014), using 3 μg of adapter per reaction (a 10:1 molar ratio). PCR amplification and hybrid capture were performed as previously described(Schmitt et al., 2015). Sequencing was performed using MiSeq V2 chemistry, using 2 × 150-bp PE reads. We then trimmed the sequences to 125 bp with Trimmomatic and placed the unique molecular identifiers into the header by using the script tag_to_header.py(Kennedy et al., 2014). Reads were aligned using BWA ALN with standard parameters. We sorted and indexed using Picard then performed consensus calling by using ConsensusMaker.py with parameters – minmem 3, –cutoff 0.8, and --Ncutoff 0.7. Unmapped reads were removed with SAMtools (http://samtools.sourceforge.net/), then local realignment was performed using GATK before creating an mpileup file. Normal and Tumor mpileup files were then compared using VarScan Somatic, with somatic mutations requiring a *P*-value of less than 10^−4^, as computed using Fisher’s exact test by VarScan (http://varscan.sourceforge.net). We also required the germline sample to have fewer than 5 reads and that no more than 90% of variant reads were on the same DNA strand. Variants underwent RefSeq annotation with ANNOVAR(Wang et al., 2010).

### Single-cell exome sequencing and mutation calling

Amplified DNA from patient 4 that had undergone single-cell isolation and whole-genome amplification using the Fluidigm C1 System as previously described was used for library construction and exome capture with the Nextera Rapid Capture Exome Kit (Illumina), used in accordance with the manufacturer’s instructions. Exome-enriched libraries then underwent sequencing using 2 ×100 reads on 4 flow cells of a HiSeq 2000 or 2500 Sequencing System (Illumina). Adapters were trimmed from each of the cells by using Trimmomatic (ILLUMINACLIP:nextera_adapters.fa:2:30:10 TRAILING:25 LEADING:25 SLIDINGWINDOW:4:20 MINLEN:30), followed by alignment with BWA using default parameters. Duplicates were marked using Picard (https://broadinstitute.github.io/picard), and local realignment followed by base score recalibration was performed using GATK (https://software.broadinstitute.org/gatk). We then called variants by using GATK and followed this with filtering using the parameter “QD < 2.0 || FS > 60.0 || MQ < 40.0 || HaplotypeScore > 13.0 || MQRankSum < −12.5 || ReadPosRankSum < −8.0”. On-target coverage was calculated with Picard HsMetrics; this was repeated after subsampling for an increasing number of reads using custom bash scripts. Custom bash scripts were also used to identify locations that had the same mutation called in more than one cell. Germline SNP locations identified by bulk sequencing were then filtered out, after which locations that were identified in any of the normal single cells were removed.

### Estimation of mutation rates

To estimate the mutation rate, we downsampled each of the files to 70 million reads. We then created marked, realigned, and base score–recalibrated BAM files as described above. This was followed by further variant calling and filtering using the GATK filtering parameters detailed above. We then subtracted those sites that were found in the bulk germline sequencing. To subtract the background error rate due to amplification errors, the somatic mosaicism rates in normal cells, and mutation miscalls, we subtracted the mean mutation rate in the 3 normal cells from that of each of the single tumor cells and plotted the distribution of the mutations rates.

### Primary cell culture and drug treatment

Primary leukemia cells were obtained from patients who had provided consent on studies approved by the St. Jude IRB. At the time of sample collection, mononuclear cells were isolated using Ficoll-Paque (GE Life Sciences) followed by cryopreservation. One vial of cells from each patient was thawed slowly using the ThawSTAR system (MedCision), and the cells were placed in culture under the conditions previously described^(Lee et al., 2023; Pieters et al., 1990)^. For the limited dilution experiment, 750,000 cells were plated in each well of a 12-well plate and were grown in culture for 3 weeks. For drug treatments, the drugs and 350,000 cells were plated in each well of a 24-well plate. In both experiments, the medium was changed twice weekly by carefully removing half of it and replacing it with fresh medium. The replacement medium included a 2× drug concentration if the cell sample was undergoing chemotherapy exposure. All drugs were purchased from Sigma-Aldrich, and the concentration ranges were based on solubility limits and previously published data^(Pieters et al., 1990)^. The drugs used were mercaptopurine (500, 250, 125, and 62.5 μg/mL ConsensusMaker.py and 90 μg/mL), vincristine (810, 162, 32.4, and 6.5 μg/mL), daunorubicin (31, 6.2, 1.2, and 0.2 μg/mL), and asparaginase (19, 9.5, 4.8, and 2.4 μg/mL). Live cells were isolated by using a dead cell removal kit (Miltenyl). DNA was extracted using a DNA Universal Kit (Zymo Research), and libraries were prepared using the HyperPlus Kit (Kapa Biosciences). Exome or custom capture was performed using oligonucleotides and the standard protocol from Integrated DNA Technologies. Quality trimming, alignment, and mutation calling were performed using the pipeline outlined above.

## Supplementary Material

Supplement 1

Supplement 2 **Table S1.** List of ALL hotspot mutation locations in the error-corrected sequencing capture panel.

Supplement 3 **Table S2.** List of recurrent mutations detected in patient SJETV077.

Supplement 4 Table S3. List of common mutations detected in relapsed samples in a previous study, highlighting genes that were also selected for in MRD cells as patients underwent treatment.

5

## Data Availability

Code used for data processing and analysis will be made available upon publication. The data are available through Mendeley Data and have been deposited in SRA at NCBI.

## Key Points

- Increasing the accuracy and resolution of sequencing from bulk to error-corrected bulk to single clone to single-cell sequencing reveals increasing levels of genetic diversity in pALL, including the presence of clone-specific driver and treatment resistance-associated mutations.
- Using PTA-enabled scWGS, single pALL cells contain several fold more mutations per cell than detected in bulk sequencing, which, when multiplied across billions of cells, reveal hidden population-scale genetic complexity.
- Some phenotypes in pALL are heritable, including treatment resistance, where we identify pre-existing rare clones with relapse-associated mutations that preferentially survive four weeks of standard treatment in patients.
