# Supplementary material for: Measuring Longitudinal Genome-wide Clonal Evolution of Pediatric Acute Lymphoblastic Leukemia at Single-Cell Resolution
Source: bioRxiv. 2025 Mar 19:2025.03.19.644196. Preprint. [Version 1] doi: 10.1101/2025.03.19.644196 (PMC11957134; doi:10.1101/2025.03.19.644196)
Supplement: Supplement 1 [file media-1.pdf]

Supplementary Methods

In vitro evolution experiment

An in vitro model of clonal evolution was developed by successively expanding and cloning single DLD-1 (ATCC CCL-221) cells over the course of four months. First, cells from the primary cell culture were expanded in RPMI-1640 with 10% fetal bovine serum for a total of 30 days. A portion of the resulting parental population was cryopreserved (population A), and the remaining cells were used to establish the first filial generation by single-cell cloning. Single-cell cloning was performed by diluting the cells in media at a concentration of <1 cell per 100uL and dispensing 100uL of the suspension into cloning cylinders. Successful clones were expanded over the course of 30 days, periodically increasing the size of the culture dish as the population grew. A portion of expanded clones (N=12) was cryopreserved and another portion was used to establish a second filial generation of cell clones. This procedure of expansion (duration 30-33 days), cryopreservation, and cloning was repeated for a total of 3 filial generations (total time 90 days). The cryopreserved population A and selected clones (N=7) from the first (B,E), second (C) and third (D,F,G,H) filial generations derived from two single cells, were thawed and independently flow sorted into 96-well plates. The isolated cells were subjected to single-cell whole-genome amplification using PTA. PTA products were fragmented and libraries were constructed from the PTA amplification product using Kapa HyperPlus workflow. Libraries resulting from two single-cell amplifications of the parental population (A1 and A2) and two single cells from each of the seven filial clones (B1, B2, E1, E2, C1, C2, D1, D2, G1, G2, F1, F2, H1 and H2) were pooled together and sequenced on a NovaSeq at the New York Genome Center, resulting in an average sequencing depth per cell higher than 30x. Sequencing data from a single-cell colony derived from the same DLD-1 cell in an independent study was downloaded to use as germline control(Póti et al. 2018).

Patient Samples

Paired diagnosis and Day 28 bone marrow mononuclear cells from pediatric B-ALL patients were collected and used in accordance with protocols approved by the Stanford University Institutional Review Board (IRB #45458) and stored at the Stanford Pediatric Bass Center Tissue Bank after obtaining informed consent. All patients were treated with standard induction therapy on Children's Oncology Group Protocols (Vincristine 2mg/m2 days 1,

Index sorting

Cryopreserved primary bone marrow mononuclear cells from B-ALL patients were thawed using a ThawStar system (BioCision), washed, and resuspended in cold PBS containing 2% FBS. Dead cells were then removed with the Dead Cell Removal Kit (Miltenyi Biotec) according to the manufacturer's protocol. After blocking with 200 µL of 5% rabbit serum (Thermo Fisher Scientific), the following antibodies were used for staining: CD45-APC-H7 (clone 2D1), CD19-APC (clone SJ25C1), CD34-APC-R700 (clone 8G12), CD38-FITC (clone T16, Beckman Coulter Life Sciences), CD10 (clone HI10A), and CD20-PE (clone L27). Minimal residual disease (MRD) blasts were selected based on the expression of CD45 and CD19. All FACS antibodies were obtained from BD Biosciences unless otherwise specified. Single $CD19^{+/-}$ $CD45^{dim}$ blast cells were sorted into 96-well plates containing 3 µL of cell buffer (BioSkryb Genomics) using the SONY SH800 index sorting module with 130 µm chips. The sorted plates were immediately placed on dry ice after sorting and stored at –80 °C until use.

Single-cell whole genome/exome sequencing

PTA was performed using the ResolveDNA Whole Genome Amplification Kit (BioSkryb Genomics) following the manufacturer's protocol. DNA from bulk samples was isolated using the QIAamp DNA Micro Kit (QIAGEN) according to the manufacturer's instructions. Whole-genome sequencing (WGS) libraries were generated using the Illumina DNA Prep with Enrichment Kit (Illumina) as per the manufacturer's protocol. Amplified libraries were selected for 200- to 500bp size using AMPure beads. Libraries were quantified and qualified using Tapestation D1000 Screen Tape (Agilent) before sequencing. Whole-exome capture was conducted using the IDT Whole Exome Panel V1.0 and IDT xGen™ Lockdown™ Reagents for Illumina's Nextera library kits. WES and WGS libraries were sequenced to 15–30× genome coverage (2×150 bp) on NovaSeq 6000(Illumina).

Initial SNV calling and phylogenetic reconstruction

WGS reads were mapped to human reference version hg38 using BWA mem (v. 0.7.17) and further processed following GATK (v. 4.1.8.1) best practices guidelines. Somatic single nucleotide variants (SNVs) were detected using Mutect2 with either the remission sample or the non-B cell fraction as matched normal. For the invitro experiment, we used the single-cell colony as control. Cell genotype likelihoods for each of the somatic positions were calculated with GATK HaplotypeCaller. Cell H3 was removed from the WGS analysis given that it was suspected of being a replicate of H4 because WGS and WES allele frequencies at exonic mutations of H3 did not match. Cells were identified as premalignant based on their lack of mutations in driver genes and absence of

chromosomal deletions. To further filter potential false positives originating during the amplification, we discarded mutations with a mean alternative allele frequency smaller than 0.35 in the cells carrying at least an alternative allele. Sites not genotyped in more than 50% of the cells were further removed. Variants were annotated with annovar using refGene, dbnsfp42c, cosmic70, avsnp150, exac03, and clinvar_20220320 as databases. Phylogenetic trees were built with CellPhy (v0.9.2) using the GT10+FO+E model. Mutations present in copy-number events shared by more than two cells were also removed prior to phylogenetic reconstruction because the CellPhy model expects diploid genotypes and then the topology can be independently validated with copy-number events.

SCAN2 analyses to determine mutation numbers and signatures

SCAN2 (Luquette et al., 2022)was independently run for each patient sample. The data resources used included the GRCh38 human reference genome, an *Eagle2* phasing panel manually created with the script *scan2_download_eagle_refpanel.sh*, and the dbSNP file downloaded from Google Cloud Storage: *dbSNP v138 for GRCh38*. Total mutational burdens for sSNVs and indels were computed separately using *mutburden.R* (*SCAN2*)for each cell. SBS96 Signature matrices were generated from VCF files using *SigProfilerMatrixGenerator* (Bergstrom et al., 2019), and cosmic signatures version 3.4 were refitted to each cell or bulk sample with *SigProfilerAssignment*(Díaz-Gay et al., 2023), excluding artifact signatures 27, 43, 45, 46, 47, 48, 49, 50, 51, 52, 53, 54, 55, 56, 57, 58, 59, 60, and 95. De novo signature extraction was performed using *SigProfilerExtractor* (Islam et al., 2022)with *maximum_signatures* set to 5.

Mutation mapping and tree calibration

Mutations were mapped to the phylogeny using treemut (https://github.com/NickWilliamsSanger/treemut). The cross-sample contamination of each cell was calculated with GATK. We hard-assigned the mutation to a given branch when it was significantly consistent with the tree (>0.05) or when the probability of being assigned to another part of the tree was null. The phylogeny was time-scaled using rtreefit (https://github.com/NickWilliamsSanger/rtreefit).

Somatic calling precision and recall calculation

Precision was calculated using a linked-read analysis approach. Bed files containing variants unique to each cell as well those on the internal branches of the tree were intersected with known heterozygous germline SNPs. Using the linked-read method, we counted the number of concordant and discordant linked-reads for each variant-hetSNP pair, while discarding reads which contained alleles at either position that did not match

our known reference or alternative alleles. Reads were filtered by edit distance, base quality, and mapping quality. If we found any discordant reads, we categorized that variant as a False Positive. Our final precision values (per-cell and overall) were calculated to be 1 - (False Discovery Rate), where the FDR was the ratio of False Positive variants to True Positives. xxx

CNV calling and AUC calculation

The germline SNPs previously detected were phased using Shapeit. In order to obtain the read counts for A and B alleles, Chisel was run. B-allele frequency (BAF) was calculated from the counts to determine if the segments carried one (loss of heterozygosity or LOH) or both haplotypes. <>. Large chromosomal gains were not detected by cytogenetics analyses, so we assumed our samples were mainly diploid. Then, the haplotype block switches were minimized in 150kb windows using a custom script to reduce the noise. <>.

Using the single-cell BAFs of hetSNPs along region chr16.q14.11 and assuming that the loss event occurred in the ancestral cells of all pink cells, we classified allelic imbalances as true positives when present in the pink cells and false positives when arising in the green cells. True negatives were counted when the green cells did not show an imbalance in a given window and false positives when they did. AUC curves were built with <>

Signature fitting

Sequence context for each mutation was extracted using a custom script. Signatures were called independently in each sample using deconstructSigs.

Phylodynamics analysis

Clonal joint posterior distributions of fitness and age of onset were inferred using Approximate Bayesian Computation (ABC). A Wright-Fisher model was used to simulate stem cell proliferation dynamics as had been previously done in *Van Egeren* (2021). Our prior on age of onset for patients was bound to be at most the age of the patient at diagnosis.

Analysis pipeline for pre and post-treatment single-cell exome sequencing

The raw sequencing data were demultiplexed using *bcl2fastq* (Illumina) with default settings. We then processed the resulting FASTQ files through *Sentieon/202112.01* (Freed et al., 2017), a set of software tools that perform analysis of genomic data obtained from DNA sequencing, which is compatible with GATK (Genome Analysis Toolkit)(McKenna et al., 2010). In more detail: Reads were trimmed with

Trimmomatic(Bolger et al., 2014)v0.35. Trimmed fastq files were aligned to the hg38 reference genome using the BWA-MEM (Md et al., 2019) aligner v0.7.17. Aligned data were sorted using *Sentieon util sort*. Duplicate reads were marked with *Sentieon LocusCollector* and removed using *Sentieon Dedup*. Indel realignment was carried out with the *Sentieon Realigner* algorithm, and base quality score recalibration (BQSR) was performed using the *Sentieon QualCal* algorithm. *Preseq* was run on downsampled 5M read BED files across different genome assemblies using the "gc_extrap" option. Alignment statistics were generated using *GATK 4.6*. The total coverage of the whole genomics was estimated with *bedtools (Quinlan and Hall, 2010) v2.30.0.* For variant analysis: Somatic variant discovery in the presence of a matched normal sample was conducted using *Sentieon TNscope*. Germline variants were called using *Sentieon Haplotyper*. Joint variant calling for germline variants was performed with *Sentieon GVCFtyper*. VCF files from the same sample were merged using *bcftools v1.1.6*, recalibrated with *GATK VariantRecalibrator*, and filtered with a 99.9 threshold. Finally, the recalibrated VCF file was annotated with annovar (Wang et al., 2010) (see code availability for details).

Phylogenetic tree construction from single cell whole exome data

The merged SNP or indel somatic variation files from the same sample were filtered based on the distribution and quality. First, variants present in more than ten cells, with an allele frequency greater than 0.1 and sequencing depth of more than four reads in at least two cells, were retained. Variants consistently observed in the majority of cells and bulk samples were excluded as germline mutations. High-quality somatic variants for phylogenetic tree construction were further filtered using the following criteria: MQRankSum = 0, MQ > 59.8, FS < 5, SOR < 3, QD between 8 and 20, and ReadPosRankSum ≥ -2. Some low-frequency variants were initially lost in bulk sample VCF files but were confirmed as true mutations in single-cell data; these variants were rescued through force calling with *Mutect2*, using *--force-active true*. The recalled VCF files were used for phylogenetic tree construction with *ConDoR* (Sashittal et al., 2023), using parameters *-a 0.001 -b 0.001 -k 5*. The tree visualization was performed using *ggtree (Yu, 2020)*, *ggplot2* (Wickham, 2016), and *aplot*.

Bergstrom, E.N., Huang, M.N., Mahto, U., Barnes, M., Stratton, M.R., Rozen, S.G., and Alexandrov, L.B. (2019). SigProfilerMatrixGenerator: a tool for visualizing and exploring patterns of small mutational events. BMC Genomics *20*, 685.
Bolger, A.M., Lohse, M., and Usadel, B. (2014). Trimmomatic: a flexible trimmer for Illumina sequence data. Bioinformatics *30*, 2114-2120.

Díaz-Gay, M., Vangara, R., Barnes, M., Wang, X., Islam, S.M.A., Vermes, I., Duke, S., Narasimman, N.B., Yang, T., Jiang, Z., *et al.* (2023). Assigning mutational signatures to individual samples and individual somatic mutations with SigProfilerAssignment. Bioinformatics *39*.

Freed, D., Aldana, R., Weber, J.A., and Edwards, J.S. (2017). The Sentieon Genomics Tools - A fast and accurate solution to variant calling from next-generation sequence data (bioRxiv), pp. https://doi.org/10.1101/115717.

Islam, S.M.A., Díaz-Gay, M., Wu, Y., Barnes, M., Vangara, R., Bergstrom, E.N., He, Y., Vella, M., Wang, J., Teague, J.W., *et al.* (2022). Uncovering novel mutational signatures by *de novo* extraction with SigProfilerExtractor. Cell Genom *2*, None.

Luquette, L.J., Miller, M.B., Zhou, Z., Bohrson, C.L., Zhao, Y., Jin, H., Gulhan, D., Ganz, J., Bizzotto, S., Kirkham, S., *et al.* (2022). Single-cell genome sequencing of human neurons identifies somatic point mutation and indel enrichment in regulatory elements. Nat Genet *54*, 1564-1571.

McKenna, A., Hanna, M., Banks, E., Sivachenko, A., Cibulskis, K., Kernytsky, A., Garimella, K., Altshuler, D., Gabriel, S., Daly, M., *et al.* (2010). The Genome Analysis Toolkit: a MapReduce framework for analyzing next-generation DNA sequencing data. Genome Res *20*, 1297-1303.

Md, V., Misra, S., Li, H., and Aluru, S. (2019). Efficient Architecture-Aware Acceleration of BWA-MEM for Multicore Systems. (*EEE Parallel and Distributed Processing Symposium (IPDPS)*).

Quinlan, A.R., and Hall, I.M. (2010). BEDTools: a flexible suite of utilities for comparing genomic features. Bioinformatics *26*, 841-842.

Sashittal, P., Zhang, H., Iacobuzio-Donahue, C.A., and Raphael, B.J. (2023). ConDoR: tumor phylogeny inference with a copy-number constrained mutation loss model. Genome Biol *24*, 272.

Wang, K., Li, M., and Hakonarson, H. (2010). ANNOVAR: functional annotation of genetic variants from high-throughput sequencing data. Nucleic Acids Res *38*, e164.

Wickham, H. (2016). ggplot2: Elegant Graphics for Data Analysis. (Springer-Verlag New York).

Yu, G. (2020). Using ggtree to Visualize Data on Tree-Like Structures. Curr Protoc Bioinformatics *69*, e96.
