# Supplementary material for: Measuring Longitudinal Genome-wide Clonal Evolution of Pediatric Acute Lymphoblastic Leukemia at Single-Cell Resolution
Source: bioRxiv. 2025 Mar 19:2025.03.19.644196. Preprint. [Version 1] doi: 10.1101/2025.03.19.644196 (PMC11957134; doi:10.1101/2025.03.19.644196)
Supplement: Supplement 2 — Table S1. List of ALL hotspot mutation locations in the error-corrected sequencing capture panel. [file media-2.pdf]

Chromosome	Start (hg19)	Stop	Gene
1	27099887	27100007	ARID1A
1	43814949	43815069	MPL
1	65310457	65310577	JAK1
1	115251185	115251305	NRAS
1	115252207	115252327	NRAS
1	115256470	115256590	NRAS
1	115258687	115258807	NRAS
10	43600440	43600560	RET
10	89692933	89693053	PTEN
10	104852901	104853021	NT5C2
10	104857030	104857150	NT5C2
11	533814	533934	HRAS
11	32417847	32417967	WT1
11	119148831	119148951	CBL
12	25362740	25362860	KRAS
12	25368346	25368466	KRAS
12	25378548	25378668	KRAS
12	25380215	25380335	KRAS
12	25398224	25398344	KRAS
12	112888150	112888270	PTPN11
13	28592582	28592702	FLT3
15	90631874	90631994	IDH2
16	3788558	3788678	CREBBP
17	7577060	7577180	TP53
17	7577478	7577598	TP53
17	7578346	7578466	TP53
18	42531847	42531967	SETBP1
19	17945909	17946029	JAK3
2	25457182	25457302	DNMT3A
2	198266774	198266894	SF3B1
2	209113052	209113172	IDH1
20	31022389	31022509	ASXL1
21	36231722	36231842	RUNX1
3	128200670	128200790	GATA2
4	55599261	55599381	KIT
4	106156687	106156807	TET2
4	153249324	153249444	FBXW7
5	35873537	35873657	IL7R
5	149433585	149433705	CSF1R
5	170837487	170837607	NPM1
7	5567936	5568056	ACTB
7	103185691	103185811	RELN
7	140453076	140453196	BRAF
7	148508667	148508787	EZH2
9	5073710	5073830	JAK2
9	133748223	133748343	ABL1
9	139390589	139390709	NOTCH1
9	139399290	139399410	NOTCH1

X	1314906	1315026	CRLF2
X	48649546	48649666	GATA1
