# Supplementary material for: Measuring Longitudinal Genome-wide Clonal Evolution of Pediatric Acute Lymphoblastic Leukemia at Single-Cell Resolution
Source: bioRxiv. 2025 Mar 19:2025.03.19.644196. Preprint. [Version 1] doi: 10.1101/2025.03.19.644196 (PMC11957134; doi:10.1101/2025.03.19.644196)
Supplement: Supplement 3 — Table S2. List of recurrent mutations detected in patient SJETV077. [file media-3.pdf]

Chromosome	Position	Reference Allele	Alternate Allele	Gene Symbol	Change Type	Base and Amino Acid Change
10	100020751	A	C	LOXL4	nonsynonymous SNV	LOXL4:NM_032211:exon4:c.T590G:p.M197R
10	102783741	C	T	PDZD7	nonsynonymous SNV	PDZD7:NM_001195263:exon3:c.G311A:p.G104D,PDZD7:NM_024895:exon3:c.G311A:p.G104D
10	25464626	C	A	GPR158	nonsynonymous SNV	GPR158:NM_020752:exon1:c.C277A:p.Q93K
10	25464650	G	A	GPR158	nonsynonymous SNV	GPR158:NM_020752:exon1:c.G301A:p.G101S
10	29843823	C	G	SVIL	nonsynonymous SNV	SVIL:NM_021738:exon5:c.G49C:p.D17H,SVIL:NM_003174:exon7:c.G49C:p.D17H
10	30918597	T	C	LYZL2	nonsynonymous SNV	LYZL2:NM_183058:exon1:c.A38G:p.K13R
10	61833362	G	A	ANK3	nonsynonymous SNV	ANK3:NM_020987:exon37:c.C7

						277T:p.S2426F
10	94828146	T	C	CYP26C1	nonsynonymous SNV	CYP26C1:NM_183374:exon6:c.T1261C:p.Y421H
10	99226266	C	G	MMS19	nonsynonymous SNV	MMS19:NM_022362:exon16:c.G1500C:p.Q500H
11	118771514	C	T	BCL9L	nonsynonymous SNV	BCL9L:NM_182557:exon6:c.G2938A:p.G980S
1	145299805	A	C	NBPF10	nonsynonymous SNV	NBPF10:NM_001039703:exon6:c.A854C:p.E285A
1	145299809	G	A	NBPF10	nonsynonymous SNV	NBPF10:NM_001039703:exon6:c.G858A:p.M286I
11	46880820	T	A	LRP4	nonsynonymous SNV	LRP4:NM_002334:exon38:c.A5432T:p.E1811V
11	47611491	T	C	C1QTNF4	nonsynonymous SNV	C1QTNF4:NM_031909:exon2:c.A872G:p.H291R
1	152327810	C	A	FLG2	nonsynonymous SNV	FLG2:NM_001014342:exon3:c.G2452T:p.A818S
11	55035844	T	C	TRIM48	nonsynonymous SNV	TRIM48:NM_024114

						:exon4:c.T574C:p.Y192H
1	156347193	G	A	RHBG	nonsynonymous SNV	RHBG:NM_020407:exon2:c.G289A:p.A97T,RHBG:NM_001256395:exon3:c.G82A:p.A28T,RHBG:NM_001256396:exon3:c.G199A:p.A67T
1	156551298	C	T	TTC24	stopgain SNV	TTC24:NM_001105669:exon2:c.C142T:p.Q48X
1	156622538	G	A	BCAN	nonsynonymous SNV	BCAN:NM_021948:exon8:c.G1796A:p.R599Q,BCAN:NM_198427:exon8:c.G1796A:p.R599Q
1	156843468	C	G	NTRK1	nonsynonymous SNV	NTRK1:NM_001012331:exon8:c.C894G:p.H298Q,NTRK1:NM_002529:exon8:c.C894G:p.H298Q,NTRK1:NM_001007792:exon9:c.C804G:p.H268Q

11	57243800	A	G	RTN4RL2	nonsynonymous SNV	RTN4RL2:NM_178570:exon3:c.A679G:p.S227G
11	57413837	A	G	YPEL4	nonsynonymous SNV	YPEL4:NM_145008:exon4:c.T227C:p.L76P
1	158641856	C	G	SPTA1	nonsynonymous SNV	SPTA1:NM_003126:exon11:c.G1481C:p.R494T
1	158641974	C	G	SPTA1	nonsynonymous SNV	SPTA1:NM_003126:exon11:c.G1363C:p.D455H
11	62296147	G	C	AHNAK	nonsynonymous SNV	AHNAK:NM_001620:exon5:c.C5742G:p.D1914E
11	62296271	G	A	AHNAK	nonsynonymous SNV	AHNAK:NM_001620:exon5:c.C5618T:p.A1873V
11	63671573	T	A	MARK2	nonsynonymous SNV	MARK2:NM_001039469:exon15:c.T1630A:p.S544T,MARK2:NM_017490:exon15:c.T1528A:p.S510T
11	63884404	A	G	FLRT1	nonsynonymous SNV	FLRT1:NM_013280:exon2:c.A665G:p.N222S

11	65321716	G	C	LTBP3	nonsynonymous SNV	LTBP3:NM_001130144:exon2:c.C467G:p.P156R,LTBP3:NM_001164266:exon2:c.C116G:p.P39R,LTBP3:NM_021070:exon2:c.C467G:p.P156R
11	65325175	G	T	LTBP3	nonsynonymous SNV	LTBP3:NM_001130144:exon1:c.C256A:p.Q86K,LTBP3:NM_021070:exon1:c.C256A:p.Q86K
1	169572299	G	A	SELP	nonsynonymous SNV	SELP:NM_003005:exon10:c.C1670T:p.S557L
1	186277070	A	G	PRG4	nonsynonymous SNV	PRG4:NM_001127710:exon4:c.A1817G:p.E606G,PRG4:NM_001127709:exon5:c.A1940G:p.E647G,PRG4:NM_001127708:exon6:c.A2096G:p.E699G,PRG4:NM_005807

						:exon7:c.A2219G:p.E740G
1	186277277	G	A	PRG4	nonsynonymous SNV	PRG4:NM_001127710:exon4:c.G2024A:p.G675E,PRG4:NM_001127709:exon5:c.G2147A:p.G716E,PRG4:NM_001127708:exon6:c.G2303A:p.G768E,PRG4:NM_005807:exon7:c.G2426A:p.G809E
1	201180415	A	G	IGFN1	nonsynonymous SNV	IGFN1:NM_001164586:exon12:c.A6394G:p.T2132A
1	201180701	G	A	IGFN1	nonsynonymous SNV	IGFN1:NM_001164586:exon12:c.G6680A:p.G2227E
1	203821508	C	G	ZC3H11A	stopgain SNV	ZC3H11A:NM_014827:exon20:c.C2414G:p.S805X
1	207787753	C	T	CR1	stopgain SNV	CR1:NM_000573:exon32:c.C5230T:p.R1744X,CR1:NM_000651:exon40:c.

						C6580T:p.R2194X
1	207787796	T	C	CR1	nonsynonymous SNV	CR1:NM_000573:exon32:c.T5273C:p.M1758T,CR1:NM_000651:exon40:c.T6623C:p.M2208T
12	108961003	C	T	ISCU	nonsynonymous SNV	ISCU:NM_213595:exon4:c.C377T:p.A126V,ISCU:NM_014301:exon5:c.C302T:p.A101V
12	109605831	C	T	ACACB	nonsynonymous SNV	ACACB:NM_001093:exon3:c.C917T:p.A306V
12	112926885	C	T	PTPN11	nonsynonymous SNV	PTPN11:NM_002834:exon13:c.C1505T:p.S502L
12	11420563	G	T	PRB3	nonsynonymous SNV	PRB3:NM_006249:exon3:c.C620A:p.P207Q
12	122839007	C	G	CLIP1	nonsynonymous SNV	CLIP1:NM_001247997:exon7:c.G1300C:p.E434Q,CLIP1:NM_002956:exon7:c.G1300C:p.E434Q,

						CLIP1:NM_198240:exon7:c.G1300C:p.E434Q
12	18435367	C	G	PIK3C2G	nonsynonymous SNV	PIK3C2G:NM_004570:exon2:c.C352G:p.Q118E
12	25378562	C	T	KRAS	nonsynonymous SNV	KRAS:NM_004985:exon4:c.G436A:p.A146T,KRAS:NM_033360:exon4:c.G436A:p.A146T
12	25398284	C	T	KRAS	nonsynonymous SNV	KRAS:NM_004985:exon2:c.G35A:p.G12D,KRAS:NM_033360:exon2:c.G35A:p.G12D
12	26276009	A	C	BHLHE41	nonsynonymous SNV	BHLHE41:NM_030762:exon5:c.T439G:p.S147A
1	226552722	G	A	PARP1	nonsynonymous SNV	PARP1:NM_001618:exon19:c.C2639T:p.A880V
12	27807681	G	C	PPFIBP1	nonsynonymous SNV	PPFIBP1:NM_001198915:exon6:c.G171C:p.L57F,PPFIBP1:NM_001198916:

						exon8:c.G630C:p.L210F,PPFIBP1:NM_003622:exon8:c.G630C:p.L210F,PPFIBP1:NM_177444:exon8:c.G630C:p.L210F
1	22987700	G	A	C1QB	nonsynonymous SNV	C1QB:NM_000491:exon3:c.G583A:p.V195I
1	22987701	T	C	C1QB	nonsynonymous SNV	C1QB:NM_000491:exon3:c.T584C:p.V195A
12	32138618	C	G	KIAA1551	nonsynonymous SNV	KIAA1551:NM_018169:exon4:c.C4729G:p.Q1577E
1	24194654	C	T	FUCA1	stopgain SNV	FUCA1:NM_000147:exon1:c.G123A:p.W41X
12	49434586	T	C	MLL2	nonsynonymous SNV	MLL2:NM_003482:exon31:c.A6967G:p.T2323A
12	49434613	T	G	MLL2	nonsynonymous SNV	MLL2:NM_003482:exon31:c.A6940C:p.T2314P
12	50746442	C	G	FAM186A	nonsynonymous SNV	FAM186A:NM_0011

						45475:exon4:c.G4173C:p.Q1391H
12	50746514	G	C	FAM186A	nonsynonymous SNV	FAM186A:NM_001145475:exon4:c.C4101G:p.H1367Q
12	50747256	T	C	FAM186A	nonsynonymous SNV	FAM186A:NM_001145475:exon4:c.A3359G:p.E1120G
12	57569740	G	A	LRP1	nonsynonymous SNV	LRP1:NM_002332:exon24:c.G3842A:p.R1281H
12	57648744	T	C	R3HDM2	nonsynonymous SNV	R3HDM2:NM_014925:exon22:c.A2743G:p.S915G
12	57919829	C	T	MBD6	nonsynonymous SNV	MBD6:NM_052897:exon6:c.C1078T:p.R360C
12	85413475	G	A	TSPAN19	nonsynonymous SNV	TSPAN19:NM_001100917:exon6:c.C380T:p.S127F
12	86373909	G	A	MGAT4C	nonsynonymous SNV	MGAT4C:NM_013244:exon7:c.C595T:p.R199C
13	25016769	G	C	PARP4	nonsynonymous SNV	PARP4:NM_006437:exon29:c.C3

						502G:p.L1168V
13	28543082	G	C	CDX2	nonsynonymous SNV	CDX2:NM_001265:exon1:c.C62G:p.S21C
13	33262654	C	T	PDS5B	nonsynonymous SNV	PDS5B:NM_015032:exon13:c.C1417T:p.R473W
13	36049631	G	C	MAB21L1	nonsynonymous SNV	MAB21L1:NM_005584:exon1:c.C645G:p.C215W
13	37011937	G	A	CCNA1	nonsynonymous SNV	CCNA1:NM_001111045:exon3:c.G466A:p.A156T,CCNA1:NM_001111046:exon3:c.G337A:p.A113T,CCNA1:NM_001111047:exon3:c.G337A:p.A113T,CCNA1:NM_003914:exon3:c.G469A:p.A157T
13	44455278	G	A	LACC1	nonsynonymous SNV	LACC1:NM_001128303:exon2:c.G157A:p.E53K,LACC1:NM_153218:exon2:c.G157A:p.E53K

13	46124044	C	T	FAM194B	nonsynonymous SNV	FAM194B:NM_182542:exon13:c.G1630A:p.E544K
1	36550650	G	A	TEKT2	nonsynonymous SNV	TEKT2:NM_014466:exon2:c.G128A:p.R43Q
1	38488443	G	A	UTP11L	nonsynonymous SNV	UTP11L:NM_016037:exon7:c.G640A:p.V214I
14	101349407	A	C	RTL1	nonsynonymous SNV	RTL1:NM_001134888:exon1:c.T1719G:p.D573E
14	105361225	G	T	CEP170B	nonsynonymous SNV	CEP170B:NM_015005:exon18:c.G4490T:p.R1497L,CEP170B:NM_001112726:exon19:c.G4595T:p.R1532L
14	105361230	A	C	CEP170B	nonsynonymous SNV	CEP170B:NM_015005:exon18:c.A4495C:p.S1499R,CEP170B:NM_001112726:exon19:c.A4600C:p.S1534R
14	21899103	C	T	CHD8	nonsynonymous SNV	CHD8:NM_001170629:exon1:c

						.G700A:p.A234T
14	23885450	G	C	MYH7	nonsynonymous SNV	MYH7:NM_000257:exon34:c.C4716G:p.I1572M
14	23899786	C	G	MYH7	nonsynonymous SNV	MYH7:NM_000257:exon11:c.G982C:p.E328Q
14	24836309	T	C	NFATC4	nonsynonymous SNV	NFATC4:NM_001136022:exon1:c.T49C:p.S17P,NFATC4:NM_001198967:exon1:c.T49C:p.S17P
14	32623912	G	A	ARHGAP5	nonsynonymous SNV	ARHGAP5:NM_001030055:exon7:c.G4267A:p.E1423K,ARHGAP5:NM_001173:exon7:c.G4264A:p.E1422K
14	62462759	C	T	SYT16	stopgain SNV	SYT16:NM_031914:exon1:c.C22T:p.Q8X
14	91633676	C	A	C14orf159	nonsynonymous SNV	C14orf159:NM_001102368:exon4:c.C211A:p.P71T,C14orf159:NM_024952:exon4:c

						.C211A:p.P71T,C14orf159:NM_001102367:exon5:c.C211A:p.P71T,C14orf159:NM_001102366:exon6:c.C211A:p.P71T,C14orf159:NM_001102369:exon6:c.C211A:p.P71T
14	92472344	G	C	TRIP11	stopgain SNV	TRIP11:NM_004239:exon11:c.C1976G:p.S659X
14	92922892	C	T	SLC24A4	nonsynonymous SNV	SLC24A4:NM_153646:exon12:c.C1195T:p.P399S,SLC24A4:NM_153648:exon12:c.C1003T:p.P335S,SLC24A4:NM_153647:exon13:c.C1138T:p.P380S
15	28483865	G	A	HERC2	nonsynonymous SNV	HERC2:NM_004667:exon24:c.C3631T:p.R1211C
15	43335458	C	T	UBR1	nonsynonymous SNV	UBR1:NM_174916:exon15:c.G1

						804A:p.E602K
15	52476793	G	C	GNB5	nonsynonymous SNV	GNB5:NM_016194:exon2:c.C81G:p.F27L
15	79748677	C	T	KIAA1024	nonsynonymous SNV	KIAA1024:NM_015206:exon2:c.C188T:p.T63M
15	85188884	C	G	WDR73	nonsynonymous SNV	WDR73:NM_032856:exon7:c.G701C:p.G234A
15	85383231	G	A	ALPK3	nonsynonymous SNV	ALPK3:NM_020778:exon5:c.G1327A:p.E443K
15	89400043	G	C	ACAN	nonsynonymous SNV	ACAN:NM_001135:exon12:c.G4227C:p.E1409D,ACAN:NM_013227:exon12:c.G4227C:p.E1409D
16	1265364	G	A	CACNA1H	nonsynonymous SNV	CACNA1H:NM_001005407:exon28:c.G5144A:p.R1715H,CACNA1H:NM_021098:exon29:c.G5162A:p.R1721H
16	1306802	A	G	TPSD1	nonsynonymous SNV	TPSD1:NM_012217:e

						xon3:c.A259G:p.I87V
16	1717967	C	T	CRAMP1L	nonsynonymous SNV	CRAMP1L:NM_020825:exon17:c.C3107T:p.S1036L
16	24834850	G	A	TNRC6A	nonsynonymous SNV	TNRC6A:NM_014494:exon25:c.G5611A:p.G1871S
16	28899031	G	A	ATP2A1	nonsynonymous SNV	ATP2A1:NM_004320:exon8:c.G916A:p.A306T,ATP2A1:NM_173201:exon8:c.G916A:p.A306T
16	3349644	G	C	TIGD7	stopgain SNV	TIGD7:NM_033208:exon2:c.C971G:p.S324X
16	3349771	G	C	TIGD7	nonsynonymous SNV	TIGD7:NM_033208:exon2:c.C844G:p.L282V
16	49670814	T	A	ZNF423	nonsynonymous SNV	ZNF423:NM_001271620:exon4:c.A2069T:p.Y690F,ZNF423:NM_015069:exon4:c.A2249T:p.Y750F
16	67271614	C	T	FHOD1	nonsynonymous SNV	FHOD1:NM_013241

						:exon7:c.G700A:p.E234K
16	67663322	G	C	CTCF	nonsynonymous SNV	CTCF:NM_001191022:exon8:c.G739C:p.D247H,CTCF:NM_006565:exon10:c.G1723C:p.D575H
16	72992206	A	C	ZFHX3	nonsynonymous SNV	ZFHX3:NM_006885:exon2:c.T1839G:p.D613E
16	72992406	T	C	ZFHX3	nonsynonymous SNV	ZFHX3:NM_006885:exon2:c.A1639G:p.T547A
16	84256031	G	A	KCNG4	nonsynonymous SNV	KCNG4:NM_172347:exon3:c.C1352T:p.T451M
16	90126823	T	G	PRDM7	nonsynonymous SNV	PRDM7:NM_001098173:exon9:c.A1159C:p.M387L
17	19607422	G	A	SLC47A2	stopgain SNV	SLC47A2:NM_001099646:exon11:c.C979T:p.Q327X,SLC47A2:NM_152908:exon11:c.C1087T:p.Q363X,SLC47A2:NM_00125666

						3:exon12:c.C1021T:p.Q341X
17	21101963	C	T	TMEM11	nonsynonymous SNV	TMEM11:NM_003876:exon2:c.G253A:p.A85T
17	27028608	G	A	SUPT6H	nonsynonymous SNV	SUPT6H:NM_003170:exon37:c.G5146A:p.D1716N
17	27085444	C	T	FAM222B	nonsynonymous SNV	FAM222B:NM_001077498:exon3:c.G1533A:p.M511I,FAM222B:NM_018182:exon4:c.G1533A:p.M511I
17	27086334	C	A	FAM222B	nonsynonymous SNV	FAM222B:NM_001077498:exon3:c.G643T:p.A215S,FAM222B:NM_018182:exon4:c.G643T:p.A215S
17	3470150	C	T	TRPV1	nonsynonymous SNV	TRPV1:NM_080706:exon15:c.G2479A:p.A827T,TRPV1:NM_018727:exon16:c.G2479A:p.A827T,TRPV1:NM_080705:e

						xon16:c.G2479A:p.A827T,TRPV1:NM_080704:exon17:c.G2479A:p.A827T
17	38249442	A	G	NR1D1,THRA	nonsynonymous SNV	NR1D1:NM_021724:exon8:c.T1739C:p.L580S,THRA:NM_001190918:exon10:c.A1163G:p.Q388R,THRA:NM_001190919:exon10:c.A1280G:p.Q427R,THRA:NM_003250:exon10:c.A1280G:p.Q427R
17	38249525	G	T	NR1D1,THRA	nonsynonymous SNV	THRA:NM_001190918:exon10:c.G1246T:p.A416S,THRA:NM_001190919:exon10:c.G1363T:p.A455S,THRA:NM_003250:exon10:c.G1363T:p.A455S
17	39274422	C	T	KRTAP4-11	nonsynonymous SNV	KRTAP4-11:NM_033059:exon

						1:c.G146A:p.C49Y
17	41246748	G	C	BRCA1	stopgain SNV	BRCA1:NM_007297:exon9:c.C659G:p.S220X,BRCA1:NM_007294:exon10:c.C800G:p.S267X,BRCA1:NM_007300:exon10:c.C800G:p.S267X
17	44895945	C	G	WNT3	nonsynonymous SNV	WNT3:NM_030753:exon1:c.G19C:p.G7R
17	48434506	C	T	XYLT2	nonsynonymous SNV	XYLT2:NM_022167:exon9:c.C1834T:p.P612S
17	48434579	G	A	XYLT2	nonsynonymous SNV	XYLT2:NM_022167:exon9:c.G1907A:p.R636H
1	75038329	G	C	C1orf173	nonsynonymous SNV	C1orf173:NM_001002912:exon14:c.C3065G:p.A1022G
17	5039172	G	A	USP6	nonsynonymous SNV	USP6:NM_004505:exon9:c.G613A:p.A205T
17	51064116	G	C	C17orf112	nonsynonymous SNV	C17orf112:NM_001243552:exo

						n3:c.G251C:p.R84T
17	7106513	G	A	DLG4	nonsynonymous SNV	DLG4:NM_001128827:exon7:c.C632T:p.A211V,DLG4:NM_001365:exon9:c.C770T:p.A257V
17	72352973	C	G	BTBD17	nonsynonymous SNV	BTBD17:NM_001080466:exon3:c.G1260C:p.Q420H
17	72352976	C	G	BTBD17	nonsynonymous SNV	BTBD17:NM_001080466:exon3:c.G1257C:p.Q419H
17	72353270	C	G	BTBD17	nonsynonymous SNV	BTBD17:NM_001080466:exon3:c.G963C:p.W321C
17	72353677	C	G	BTBD17	nonsynonymous SNV	BTBD17:NM_001080466:exon3:c.G556C:p.E186Q
17	72353701	C	T	BTBD17	nonsynonymous SNV	BTBD17:NM_001080466:exon3:c.G532A:p.G178S
17	72916548	T	C	USH1G	nonsynonymous SNV	USH1G:NM_173477:exon2:c.A383G:p.N128S
17	7577566	T	C	TP53	nonsynonymous SNV	TP53:NM_001126115:exon3:c.

A319G:p.N
107D,TP53
:NM_0011
26116:exo
n3:c.A319
G:p.N107D
,TP53:NM
_0011261
17:exon3:c
.A319G:p.
N107D,TP
53:NM_00
1276697:e
xon3:c.A2
38G:p.N80
D,TP53:N
M_001276
698:exon3
:c.A238G:p
.N80D,TP5
3:NM_001
276699:ex
on3:c.A23
8G:p.N80D
,TP53:NM
_0011261
18:exon6:c
.A598G:p.
N200D,TP
53:NM_00
0546:exon
7:c.A715G:
p.N239D,T
P53:NM_0
01126112:
exon7:c.A
715G:p.N2
39D,TP53:
NM_0011
26113:exo
n7:c.A715
G:p.N239D
,TP53:NM
_0011261

						14:exon7:c.A715G:p.N239D,TP53:NM_001276695:exon7:c.A598G:p.N200D,TP53:NM_001276696:exon7:c.A598G:p.N200D,TP53:NM_001276760:exon7:c.A598G:p.N200D,TP53:NM_001276761:exon7:c.A598G:p.N200D
17	7579374	C	A	TP53	nonsynonymous SNV	TP53:NM_001126118:exon3:c.G196T:p.G66C,TP53:NM_000546:exon4:c.G313T:p.G105C,TP53:NM_001126112:exon4:c.G313T:p.G105C,TP53:NM_001126113:exon4:c.G313T:p.G105C,TP53:NM_001126114:exon4:c.G313T:p.G105C,TP53:N

						M_001276695:exon4:c.G196T:p.G66C,TP53:NM_001276696:exon4:c.G196T:p.G66C,TP53:NM_001276760:exon4:c.G196T:p.G66C,TP53:NM_001276761:exon4:c.G196T:p.G66C
1	86047232	G	A	CYR61	nonsynonymous SNV	CYR61:NM_001554:exon2:c.G248A:p.S83N
18	6986227	G	A	LAMA1	nonsynonymous SNV	LAMA1:NM_005559:exon37:c.C5288T:p.A1763V
2	107049414	G	A	RGPD3	nonsynonymous SNV	RGPD3:NM_001144013:exon17:c.C2446T:p.R816C
2	109367844	T	G	RANBP2	nonsynonymous SNV	RANBP2:NM_006267:exon10:c.T1398G:p.H466Q
2	114674544	A	G	ACTR3	nonsynonymous SNV	ACTR3:NM_001277140:exon3:c.A31G:p.I11V,ACTR3:NM_005721:exon3:c

						.A184G:p.I62V
2	132240363	A	G	TUBA3D	nonsynonymous SNV	TUBA3D:NM_080386:exon5:c.A1295G:p.Y432C
2	144381810	T	C	ARHGAP15	nonsynonymous SNV	ARHGAP15:NM_018460:exon12:c.T1112C:p.F371S
2	144381811	C	G	ARHGAP15	nonsynonymous SNV	ARHGAP15:NM_018460:exon12:c.C1113G:p.F371L
2	162895664	C	T	DPP4	nonsynonymous SNV	DPP4:NM_001935:exon6:c.G397A:p.D133N
2	17699517	G	T	RAD51AP2	nonsynonymous SNV	RAD51AP2:NM_001099218:exon1:c.C166A:p.R56S
2	186668077	G	A	FSIP2	nonsynonymous SNV	FSIP2:NM_173651:exon17:c.G14311A:p.E4771K
2	198363406	C	T	HSPD1	nonsynonymous SNV	HSPD1:NM_002156:exon2:c.G167A:p.G56E,HSPD1:NM_199440:exon2:c.G167A:p.G56E
2	207605813	A	T	MDH1B	nonsynonymous SNV	MDH1B:NM_001039845:exon1

						0:c.T1424A:p.V475D
2	220082421	C	A	ABCB6	stopgain SNV	ABCB6:NM_005689:exon2:c.G658T:p.E220X
2	220315893	G	A	SPEG	nonsynonymous SNV	SPEG:NM_005876:exon5:c.G2149A:p.E717K
2	26689656	A	G	OTOF	nonsynonymous SNV	OTOF:NM_194322:exon18:c.T2356C:p.S786P,OTOF:NM_004802:exon19:c.T2125C:p.S709P,OTOF:NM_194323:exon19:c.T2125C:p.S709P,OTOF:NM_194248:exon36:c.T4426C:p.S1476P
2	27282122	A	T	AGBL5	nonsynonymous SNV	AGBL5:NM_001035507:exon11:c.A1939T:p.S647C,AGBL5:NM_021831:exon11:c.A1939T:p.S647C
2	27601820	C	T	ZNF513	nonsynonymous SNV	ZNF513:NM_001201459:exon2:c.G127A:p

						.A43T,ZNF513:NM_144631:exon3:c.G313A:p.A105T
2	73302692	G	C	RAB11FIP5	nonsynonymous SNV	RAB11FIP5:NM_015470:exon5:c.C1919G:p.T640S
2	75101564	C	T	HK2	nonsynonymous SNV	HK2:NM_000189:exon7:c.C863T:p.P288L
2	88082426	C	A	RGPD1,RGPD2	nonsynonymous SNV	RGPD1:NM_001024457:exon20:c.G4093T:p.D1365Y,RGPD2:NM_001078170:exon20:c.G4117T:p.D1373Y
3	102196305	C	T	ZPLD1	nonsynonymous SNV	ZPLD1:NM_175056:exon11:c.C1139T:p.P380L
3	127783850	C	G	SEC61A1	nonsynonymous SNV	SEC61A1:NM_013336:exon8:c.C747G:p.I249M
3	130799347	C	T	NEK11	nonsynonymous SNV	NEK11:NM_001146003:exon3:c.C251T:p.S84F,NEK11:NM_024800:exon4:c.C251T:p.S84F,NEK11

						:NM_145910:exon4:c.C251T:p.S84F
3	168833952	A	G	MECOM	nonsynonymous SNV	MECOM:NM_001105078:exon7:c.T1144C:p.F382L,MECOM:NM_001163999:exon7:c.T1147C:p.F383L,MECOM:NM_001164000:exon7:c.T1144C:p.F382L,MECOM:NM_001205194:exon7:c.T1144C:p.F382L,MECOM:NM_005241:exon7:c.T1144C:p.F382L,MECOM:NM_001105077:exon8:c.T1339C:p.F447L,MECOM:NM_004991:exon8:c.T1708C:p.F570L
3	168834012	A	G	MECOM	nonsynonymous SNV	MECOM:NM_001105078:exon7:c.T1084C:p.S362P,MECOM:NM

						_001163999:exon7:c.T1087C:p.S363P,MECOM:NM_001164000:exon7:c.T1084C:p.S362P,MECOM:NM_001205194:exon7:c.T1084C:p.S362P,MECOM:NM_005241:exon7:c.T1084C:p.S362P,MECOM:NM_001105077:exon8:c.T1279C:p.S427P,MECOM:NM_004991:exon8:c.T1648C:p.S550P
3	183368618	C	G	KLHL24	nonsynonymous SNV	KLHL24:NM_017644:exon3:c.C474G:p.F158L
3	183689545	A	C	ABCC5	nonsynonymous SNV	ABCC5:NM_005688:exon11:c.T1567G:p.S523A
3	32031962	A	G	ZNF860	nonsynonymous SNV	ZNF860:NM_001137674:exon2:c.A1391G:p.H464R

3	32032046	G	A	ZNF860	nonsynonymous SNV	ZNF860:NM_001137674:exon2:c.G1475A:p.R492H
3	33155909	C	T	CRTAP	nonsynonymous SNV	CRTAP:NM_006371:exon1:c.C340T:p.R114C
3	57443708	C	T	DNAH12	nonsynonymous SNV	DNAH12:NM_178504:exon22:c.G3007A:p.E1003K
4	100234991	C	T	ADH1B	nonsynonymous SNV	ADH1B:NM_000668:exon6:c.G815A:p.R272Q
4	146062683	A	G	OTUD4	nonsynonymous SNV	OTUD4:NM_001102653:exon19:c.T1736C:p.I579T
4	15004680	T	A	CPEB2	nonsynonymous SNV	CPEB2:NM_001177381:exon1:c.T383A:p.I128N,CPEB2:NM_001177382:exon1:c.T383A:p.I128N,CPEB2:NM_001177383:exon1:c.T383A:p.I128N,CPEB2:NM_001177384:exon1:c.T383A:p.I128N,CPEB2:NM

						_182485:exon1:c.T383A:p.I128N,CPEB2:NM_182646:exon1:c.T383A:p.I128N
4	15004758	C	G	CPEB2	nonsynonymous SNV	CPEB2:NM_001177381:exon1:c.C461G:p.S154C,CPEB2:NM_001177382:exon1:c.C461G:p.S154C,CPEB2:NM_001177383:exon1:c.C461G:p.S154C,CPEB2:NM_001177384:exon1:c.C461G:p.S154C,CPEB2:NM_182485:exon1:c.C461G:p.S154C,CPEB2:NM_182646:exon1:c.C461G:p.S154C
4	1918753	G	A	WHSC1	nonsynonymous SNV	WHSC1:NM_133334:exon3:c.G916A:p.E306K,WHSC1:NM_001042424:exon4:c.G916A:p.E306

						K,WHSC1:NM_133335:exon4:c.G916A:p.E306K,WHSC1:NM_133331:exon5:c.G916A:p.E306K,WHSC1:NM_007331:exon6:c.G916A:p.E306K,WHSC1:NM_133330:exon6:c.G916A:p.E306K
4	1920348	G	A	WHSC1	nonsynonymous SNV	WHSC1:NM_133334:exon4:c.G1408A:p.E470K,WHSC1:NM_001042424:exon5:c.G1408A:p.E470K,WHSC1:NM_133335:exon5:c.G1408A:p.E470K,WHSC1:NM_133331:exon6:c.G1408A:p.E470K,WHSC1:NM_007331:exon7:c.G1408A:p.E470K,WHSC1:NM_133330:exon7:c.G14

						08A:p.E470K
4	3254966	C	T	MSANTD1	nonsynonymous SNV	MSANTD1:NM_001042690:exon2:c.C353T:p.A118V
4	3255001	G	A	MSANTD1	nonsynonymous SNV	MSANTD1:NM_001042690:exon2:c.G388A:p.G130R
4	99355110	G	C	RAP1GDS1	nonsynonymous SNV	RAP1GDS1:NM_001100430:exon11:c.G1191C:p.Q397H,RAP1GDS1:NM_001100428:exon12:c.G1320C:p.Q440H,RAP1GDS1:NM_001100429:exon12:c.G1317C:p.Q439H,RAP1GDS1:NM_001100426:exon13:c.G1467C:p.Q489H,RAP1GDS1:NM_001100427:exon13:c.G1464C:p.Q488H,RAP1GDS1:NM_021159:exon13:c.G1464C:p.Q488H

5	112151253	C	G	APC	nonsynonymous SNV	APC:NM_001127511:exon7:c.C842G:p.S281C,APC:NM_000038:exon9:c.C896G:p.S299C,APC:NM_001127510:exon10:c.C896G:p.S299C
5	140250188	C	G	PCDHA11	nonsynonymous SNV	PCDHA11:NM_018902:exon1:c.C1500G:p.D500E,PCDHA11:NM_031861:exon1:c.C1500G:p.D500E
5	140517305	C	G	PCDHB5	nonsynonymous SNV	PCDHB5:NM_015669:exon1:c.C2289G:p.F763L
5	140812343	G	A	PCDHGA12	nonsynonymous SNV	PCDHGA12:NM_003735:exon1:c.G2017A:p.A673T,PCDHGA12:NM_032094:exon1:c.G2017A:p.A673T
5	157170792	G	C	LSM11	nonsynonymous SNV	LSM11:NM_173491:exon1:c.G34C:p.G12R
5	159545858	A	G	PWWP2A	nonsynonymous SNV	PWWP2A:NM_0011

						30864:exon1:c.T538C:p.F180L,PWWP2A:NM_001267035:exon1:c.T538C:p.F180L,PWWP2A:NM_052927:exon1:c.T538C:p.F180L
5	228322	A	G	SDHA	nonsynonymous SNV	SDHA:NM_004168:exon6:c.A644G:p.Y215C
5	23527167	G	C	PRDM9	nonsynonymous SNV	PRDM9:NM_020227:exon11:c.G1970C:p.R657T
5	23527472	G	A	PRDM9	nonsynonymous SNV	PRDM9:NM_020227:exon11:c.G2275A:p.D759N
5	23527637	A	C	PRDM9	nonsynonymous SNV	PRDM9:NM_020227:exon11:c.A2440C:p.S814R
5	23527724	A	G	PRDM9	nonsynonymous SNV	PRDM9:NM_020227:exon11:c.A2527G:p.N843D
5	32089885	G	A	PDZD2	nonsynonymous SNV	PDZD2:NM_178140:exon19:c.G6331A:p.E2111K
5	40692166	C	A	PTGER4	nonsynonymous SNV	PTGER4:NM_000958

						:exon3:c.C1153A:p.L385M
5	52365978	C	T	ITGA2	nonsynonymous SNV	ITGA2:NM_002203:exon17:c.C2123T:p.S708L
5	79616400	G	A	SPZ1	nonsynonymous SNV	SPZ1:NM_032567:exon1:c.G366A:p.M122I
6	107955780	A	G	SOBP	nonsynonymous SNV	SOBP:NM_018013:exon6:c.A1732G:p.S578G
6	12126032	C	G	HIVEP1	nonsynonymous SNV	HIVEP1:NM_002114:exon4:c.C6004G:p.Q2002E
6	1390534	A	G	FOXF2	nonsynonymous SNV	FOXF2:NM_001452:exon1:c.A352G:p.S118G
6	1390535	G	C	FOXF2	nonsynonymous SNV	FOXF2:NM_001452:exon1:c.G353C:p.S118T
6	168377119	T	C	HGC6.3	nonsynonymous SNV	HGC6.3:NM_001129895:exon1:c.A214G:p.M72V
6	168430272	T	A	KIF25	nonsynonymous SNV	KIF25:NM_005355:exon2:c.T7A:p.W3R,KIF25:NM_030615:exon

						2:c.T7A:p.W3R
6	31605302	G	A	PRRC2A	nonsynonymous SNV	PRRC2A:NM_004638:exon31:c.G6413A:p.R2138Q,PRRC2A:NM_080686:exon31:c.G6413A:p.R2138Q
6	33415660	G	A	SYNGAP1	nonsynonymous SNV	SYNGAP1:NM_006772:exon18:c.G3835A:p.A1279T
6	6002600	G	C	NRN1	nonsynonymous SNV	NRN1:NM_016588:exon2:c.C186G:p.I62M
6	89895130	T	C	GABRR1	nonsynonymous SNV	GABRR1:NM_001256703:exon6:c.A644G:p.K215R,GABRR1:NM_002042:exon7:c.A695G:p.K232R,GABRR1:NM_001256704:exon8:c.A434G:p.K145R,GABRR1:NM_001267582:exon9:c.A434G:p.K145R
7	100482090	G	C	SRRT	nonsynonymous SNV	SRRT:NM_001128852:exon7:c.G859C:p.E

						287Q,SRRT:NM_001128853:exon7:c.G859C:p.E287Q,SRRT:NM_001128854:exon7:c.G859C:p.E287Q,SRRT:NM_015908:exon7:c.G859C:p.E287Q
7	105254947	G	C	ATXN7L1	nonsynonymous SNV	ATXN7L1:NM_138495:exon8:c.C1462G:p.P488A,ATXN7L1:NM_020725:exon10:c.C1834G:p.P612A
7	105254977	A	G	ATXN7L1	nonsynonymous SNV	ATXN7L1:NM_138495:exon8:c.T1432C:p.S478P,ATXN7L1:NM_020725:exon10:c.T1804C:p.S602P
7	150094143	T	C	ZNF775	nonsynonymous SNV	ZNF775:NM_173680:exon3:c.T574C:p.S192P
7	150768907	G	C	SLC4A2	nonsynonymous SNV	SLC4A2:NM_001199693:exon14:c.G2296C:p.E766Q,

						SLC4A2:NM_001199694:exon14:c.G2281C:p.E761Q,SLC4A2:NM_001199692:exon15:c.G2323C:p.E775Q,SLC4A2:NM_003040:exon15:c.G2323C:p.E775Q
7	151962134	G	T	MLL3	stopgain SNV	MLL3:NM_170606:exon8:c.C1173A:p.C391X
7	2581772	G	C	BRAT1	nonsynonymous SNV	BRAT1:NM_152743:exon7:c.C997G:p.Q333E
7	27238961	T	C	HOXA13	nonsynonymous SNV	HOXA13:NM_000522:exon1:c.A736G:p.M246V
7	27238988	C	G	HOXA13	nonsynonymous SNV	HOXA13:NM_000522:exon1:c.G709C:p.A237P
7	33044951	C	G	FKBP9	nonsynonymous SNV	FKBP9:NM_007270:exon10:c.C1701G:p.H567Q
7	33044951	C	T	FKBP9	synonymous SNV	FKBP9:NM_007270:exon10:c.C1

						701T:p.H567H
7	57187725	G	T	ZNF479	nonsynonymous SNV	ZNF479:NM_033273:exon5:c.C1397A:p.T466K
7	57187780	A	C	ZNF479	nonsynonymous SNV	ZNF479:NM_033273:exon5:c.T1342G:p.L448V
7	75613144	G	A	POR	nonsynonymous SNV	POR:NM_000941:exon10:c.G1036A:p.D346N
7	89790570	G	C	STEAP1	nonsynonymous SNV	STEAP1:NM_012449:exon3:c.G536C:p.S179T
8	143695347	A	T	ARC	nonsynonymous SNV	ARC:NM_015193:exon1:c.T286A:p.C96S
8	145059786	T	A	PARP10	nonsynonymous SNV	PARP10:NM_032789:exon4:c.A467T:p.N156I
8	26372007	T	A	DPYSL2	nonsynonymous SNV	DPYSL2:NM_001197293:exon1:c.T166A:p.S56T
9	113341430	A	T	SVEP1	nonsynonymous SNV	SVEP1:NM_153366:exon1:c.T394A:p.Y132N
9	12702433	T	C	TYRP1	nonsynonymous SNV	TYRP1:NM_000550:exon5:c.T10

						76C:p.V359A
9	133944381	A	T	LAMC3	nonsynonymous SNV	LAMC3:NM_006059:exon16:c.A2834T:p.Q945L
9	135139870	A	T	SETX	nonsynonymous SNV	SETX:NM_015046:exon26:c.T7790A:p.L2597Q
9	135946015	T	C	CEL	nonsynonymous SNV	CEL:NM_001807:exon10:c.T1463C:p.I488T
9	139740877	C	G	C9orf172	nonsynonymous SNV	C9orf172:NM_001080482:exon1:c.C2011G:p.R671G
9	139992338	G	A	MAN1B1	nonsynonymous SNV	MAN1B1:NM_016219:exon5:c.G679A:p.E227K
9	140080774	C	T	ANAPC2	nonsynonymous SNV	ANAPC2:NM_013366:exon3:c.G775A:p.E259K
9	34089478	C	G	DCAF12	nonsynonymous SNV	DCAF12:NM_015397:exon8:c.G1135C:p.A379P
9	34990713	C	T	DNAJB5	nonsynonymous SNV	DNAJB5:NM_001135005:exon1:c.C86T:p.S29F,DNAJB5:NM_001135004:ex

on2:c.C21
2T:p.S71F
