# Supplementary material for: Measuring Longitudinal Genome-wide Clonal Evolution of Pediatric Acute Lymphoblastic Leukemia at Single-Cell Resolution
Source: bioRxiv. 2025 Mar 19:2025.03.19.644196. Preprint. [Version 1] doi: 10.1101/2025.03.19.644196 (PMC11957134; doi:10.1101/2025.03.19.644196)
Supplement: 5 [file NIHPP2025.03.19.644196V1-supplement-1.pdf]

bioRxiv preprint doi: https://doi.org/10.1101/2025.03.19.644196; this version posted March 19, 2025. The copyright holder for this preprint (which was not certified by peer review) is the author/funder, who has granted bioRxiv a license to display the preprint in perpetuity. It is made available under aCC-BY 4.0 International license.

SUPPLEMENTARY MATERIALS

Figure S1. Distribution of duplicate numbers in error-corrected sequencing. Unique molecular identifier family size distributions for (A) germline and (B) leukemia samples.

Figure S1. Saturation of sequencing coverage at increasing depth. (A) Exome sequencing of bulk samples reached a saturating coverage breadth of 90% with 10X

bioRxiv preprint doi: https://doi.org/10.1101/2025.03.19.644196; this version posted March 19, 2025. The copyright holder for this preprint (which was not certified by peer review) is the author/funder, who has granted bioRxiv a license to display the preprint in perpetuity. It is made available under aCC-BY 4.0 International license.

coverage at 40 million reads. (B) MDA single cells reached a saturating 10X coverage breadth of 55% at 60 million reads.

Figure S3. Comparing PTA and MDA exome sequencing at increasing number of reads (A) Fold enrichment (B) Heterozygous SNP sensitivity (C) Coverage at increased depth.

Figure S4. Additional ETV6-RUNX1 pALL PTA-based WGS single cell metrics (A) Mutations in bulk and single cells expressed as mutations per Mb (B) Evaluation of genome coverage and variant density for bulk and single cells for additional genomic locations. (D-G) Known SBS signatures that were detected in each sample and single cell, including shared and unique mutations. This is in comparison to the de novo mutation discovery used in Figure 3.

Figure S5. Supplementary PTA scWGS Performance Metrics (A) Coverage Median Absolute Deviation (MAD) in neighboring 10Mb-long genomic bins for a different set of samples. Coverage was calculated after downsampling the files at 0.1x. Violin plots are shown when there was more than 1 sample per dataset. Violin plots include mean and standard error bars calculated by computing non-parametric bootstrap. (B) Single-cell coverage breadth at different sequencing efforts for 16 PTA-amplified DLD1 cells. Some library were sequenced twice on a NovaSeq platform. When the same library has been sequenced twice we added the value from the first run and from the combination of the two runs. (C-D). B-allele frequency at germline heterozygous SNPs in chromosome 21 for a PTA-amplified DLD1 cell (C) and for a MDA-amplified NA12878 cell (D). Germline

bioRxiv preprint doi: https://doi.org/10.1101/2025.03.19.644196; this version posted March 19, 2025. The copyright holder for this preprint (which was not certified by peer review) is the author/funder, who has granted bioRxiv a license to display the preprint in perpetuity. It is made available under aCC-BY 4.0 International license.

SNPS for the PTA-amplified cell were detected following GATK best practices on a DLD1 single-cell colony. Germline SNPs for the MDA-amplified cell were downloaded from the 1000 Genomes Project. Reads were downsampled in silico and BAF was assessed at any position showing at least 1 read. (E) Allelic dropout (ADO) or percentage of heterozygous germline sites showing only one allele while covered by at least 15 reads. (F) Recall by coverage based on the number of sites in which we obtain the variant allele at heterozygous germline sites. (G) Histogram of the B-allele frequency for the sites showing at least one read along chromosome 21.

Figure S6. Additional details, metrics, and validation of single cell variant calling strategy (A) Experimental design and pipeline applied to build phenotypically-annotated PTA phylogenies (B) Precision and recall value ranges provided in external publications and for own data (phycall). (C) Tree representing the possible cell histories under the designed in-vitro evolutionary experiment. Two cells were sampled from the established parental cell population (A1 and A2) whereas the remaining fourteen cells were isolated from seven different single-cell clones from the filial generations. Politomies represent uncertainty in node order given the experimental set up. (D) B-allele frequency of the cells within the patient A phylogeny for the somatic mutations detected in the whole-exome sequencing data. (E) PCA of the fluorescence intensity of the 6 flow-cell markers recorded for the cells within the patient B phylogeny. (F) PCA of the fluorescence intensity of the 6 flow-cell markers recorded for the cells within the patient C phylogeny. (G) Phylodyn analysis for the tumor populations in patients B and C.

Figure S1

A)

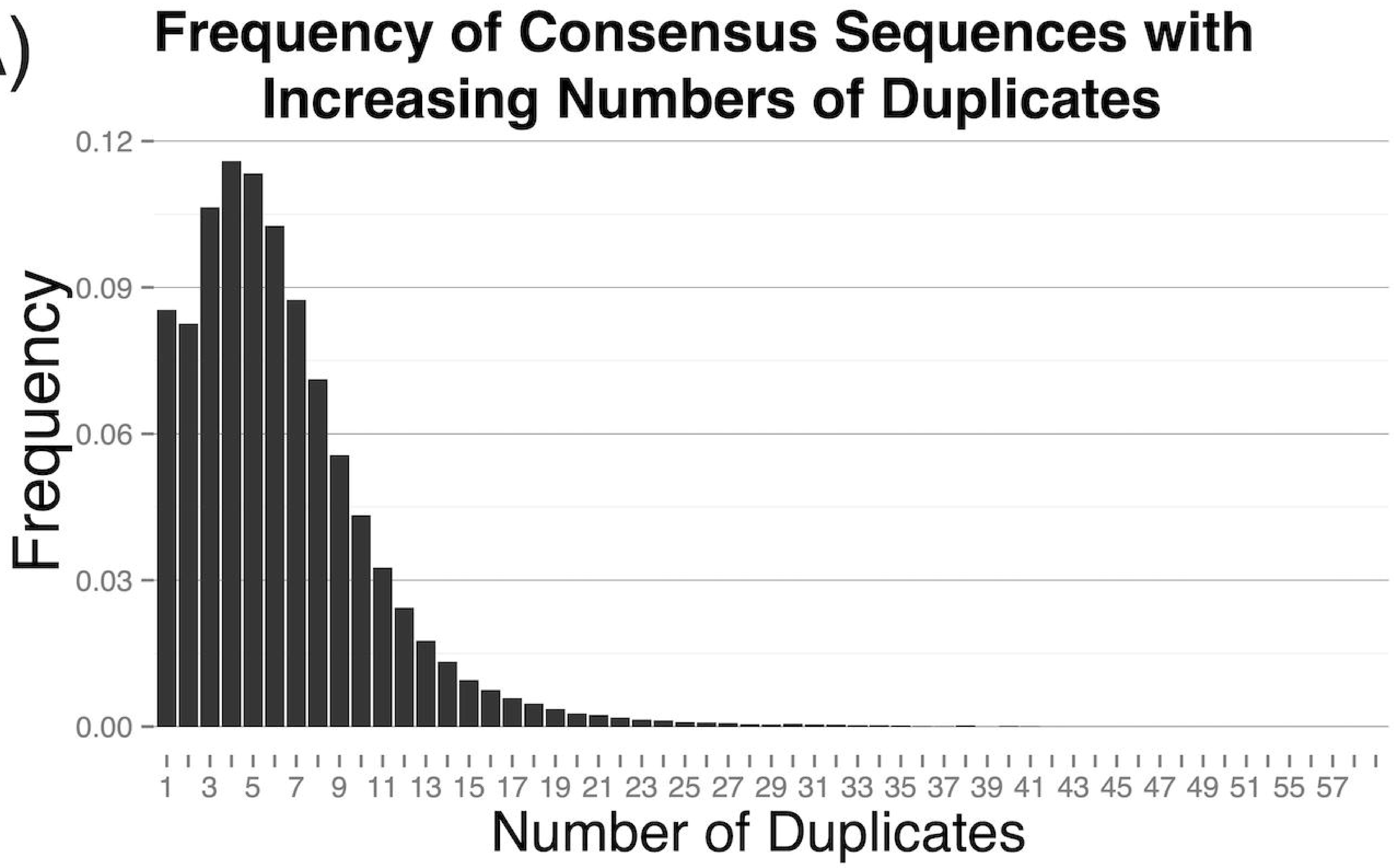

B)

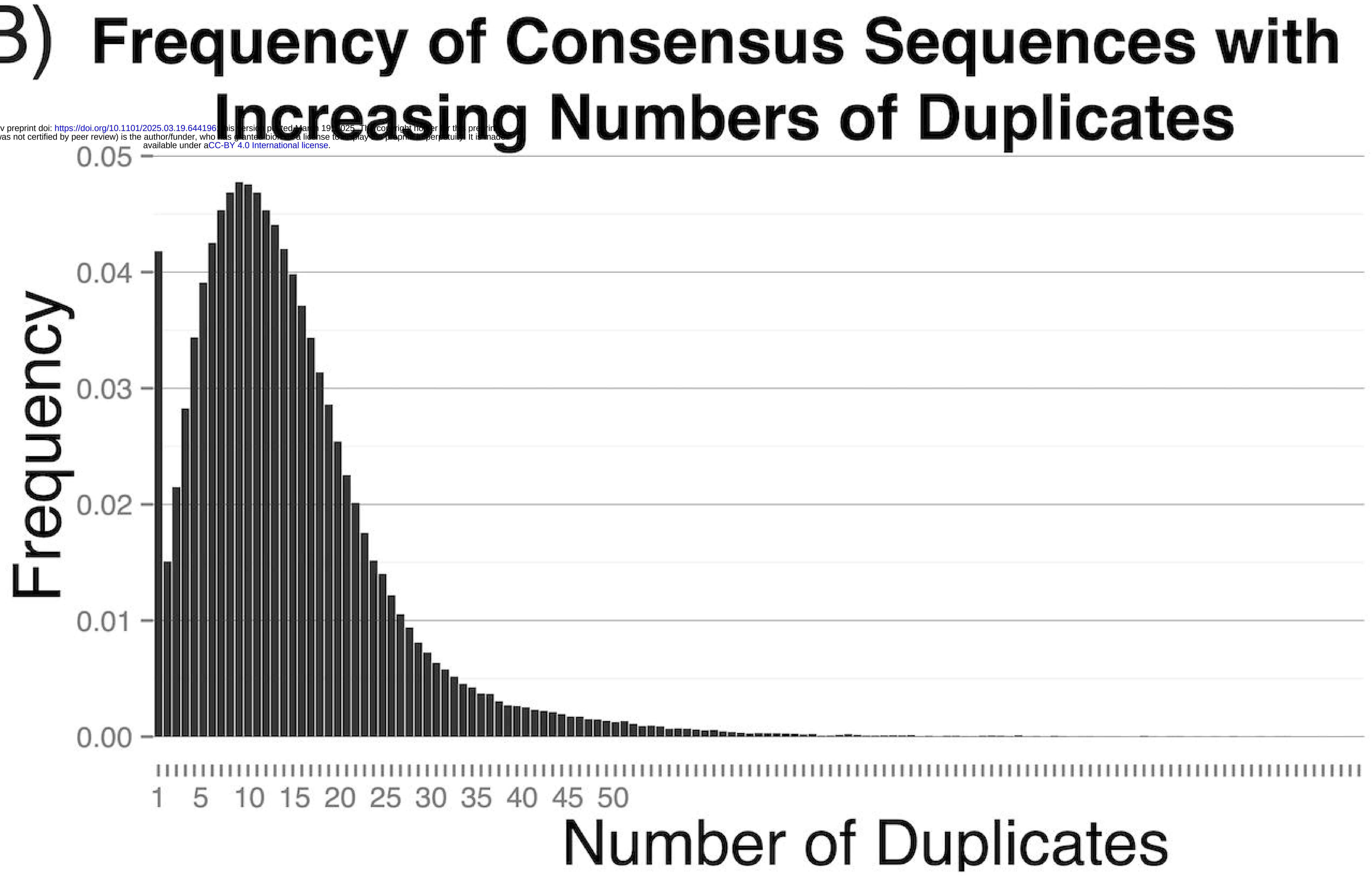

bioRxiv preprint doi: https://doi.org/10.1101/2025.03.19.644196; this version posted March 19, 2025. The copyright holder for this preprint (which was not certified by peer review) is the author/funder, who has granted bioRxiv a license to display the preprint in perpetuity. It is made available under aCC-BY 4.0 International license.

Figure S2

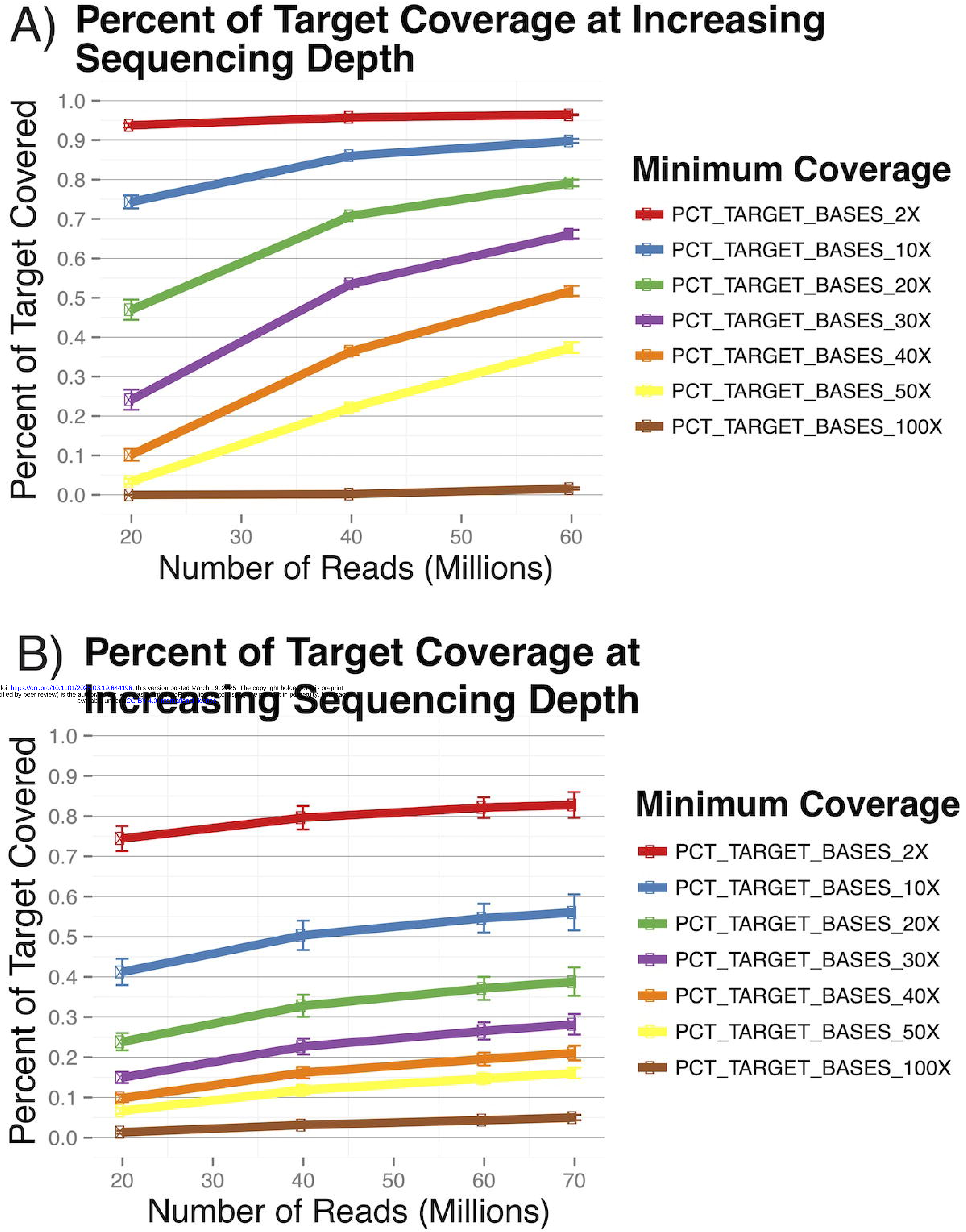

bioRxiv preprint doi: https://doi.org/10.1101/2025.03.19.644196; this version posted March 19, 2025. The copyright holder for this preprint (which was not certified by peer review) is the author/funder, who has granted bioRxiv a license to display the preprint in perpetuity. It is made available under aCC-BY 4.0 International license.

Figure S3

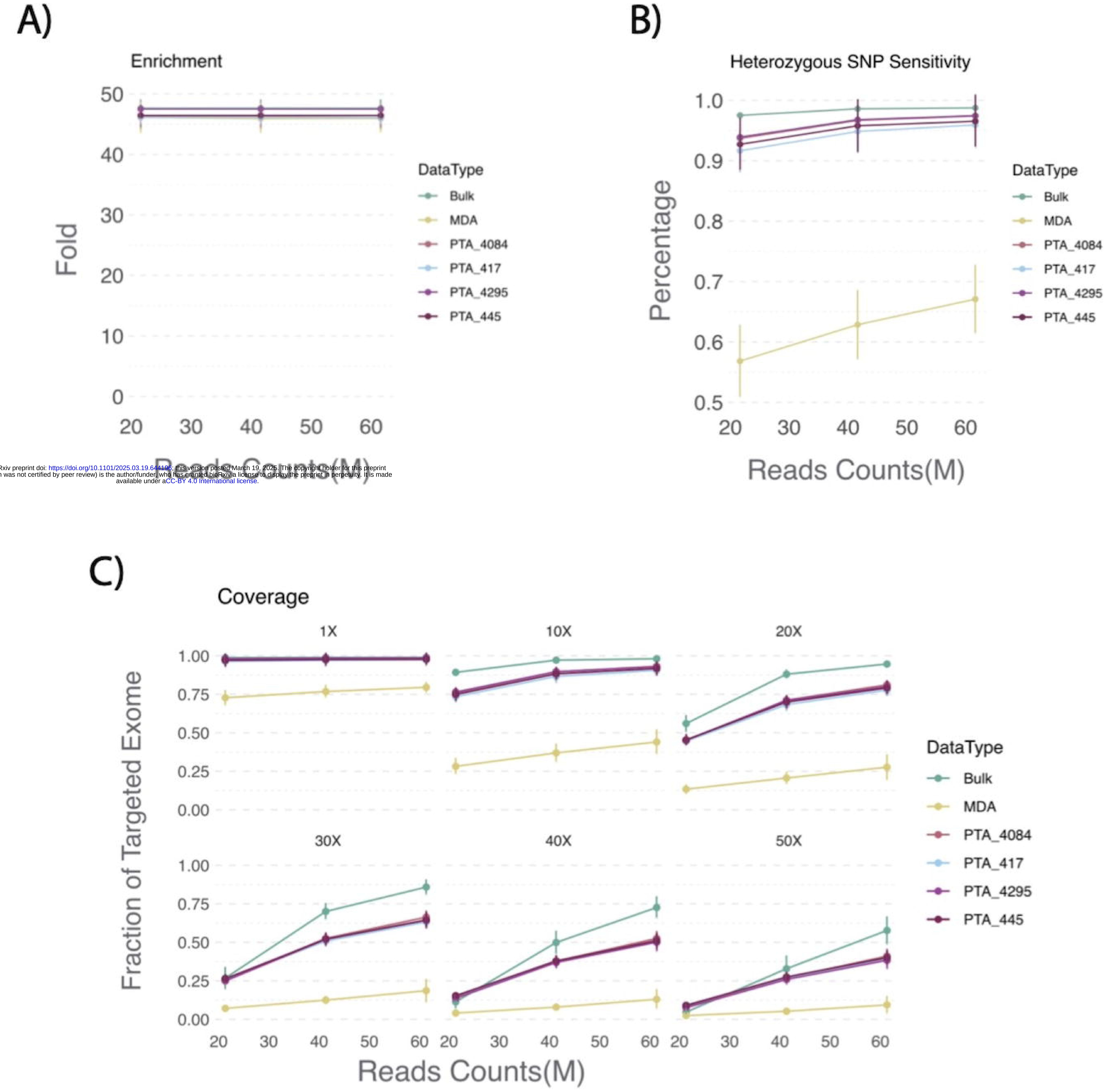

bioRxiv preprint doi: https://doi.org/10.1101/2025.03.19.644196; this version posted March 19, 2025. The copyright holder for this preprint (which was not certified by peer review) is the author/funder, who has granted bioRxiv a license to display the preprint in perpetuity. It is made available under aCC-BY 4.0 International license.

Figure S4

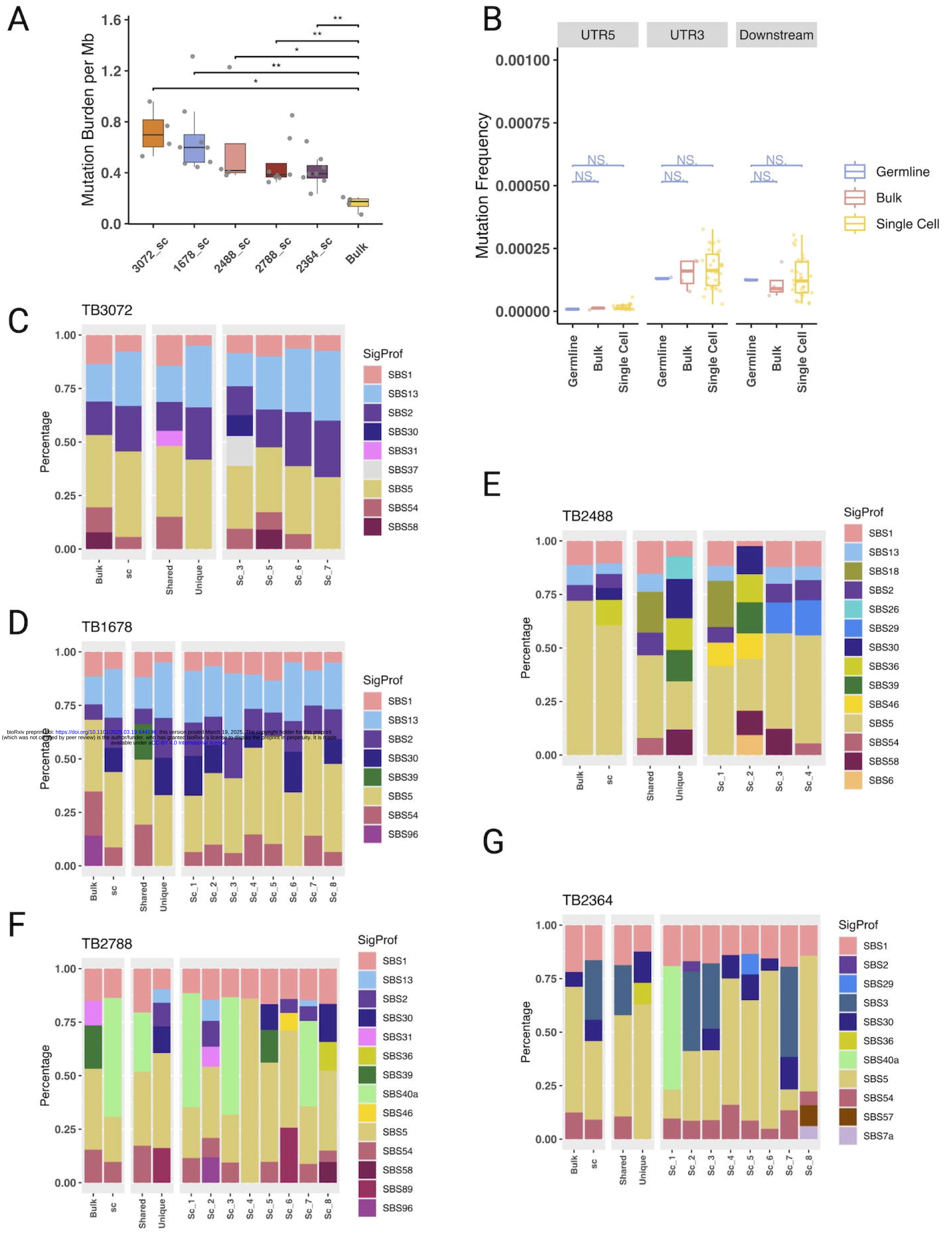

bioRxiv preprint doi: https://doi.org/10.1101/2025.03.19.644196; this version posted March 19, 2025. The copyright holder for this preprint (which was not certified by peer review) is the author/funder, who has granted bioRxiv a license to display the preprint in perpetuity. It is made available under aCC-BY 4.0 International license.

(which was not certified by peer review) is the author/funder, who has granted bioRxiv a license to display the preprint in perpetuity. It is made available under aCC-BY 4.0 International license.

Figure S5

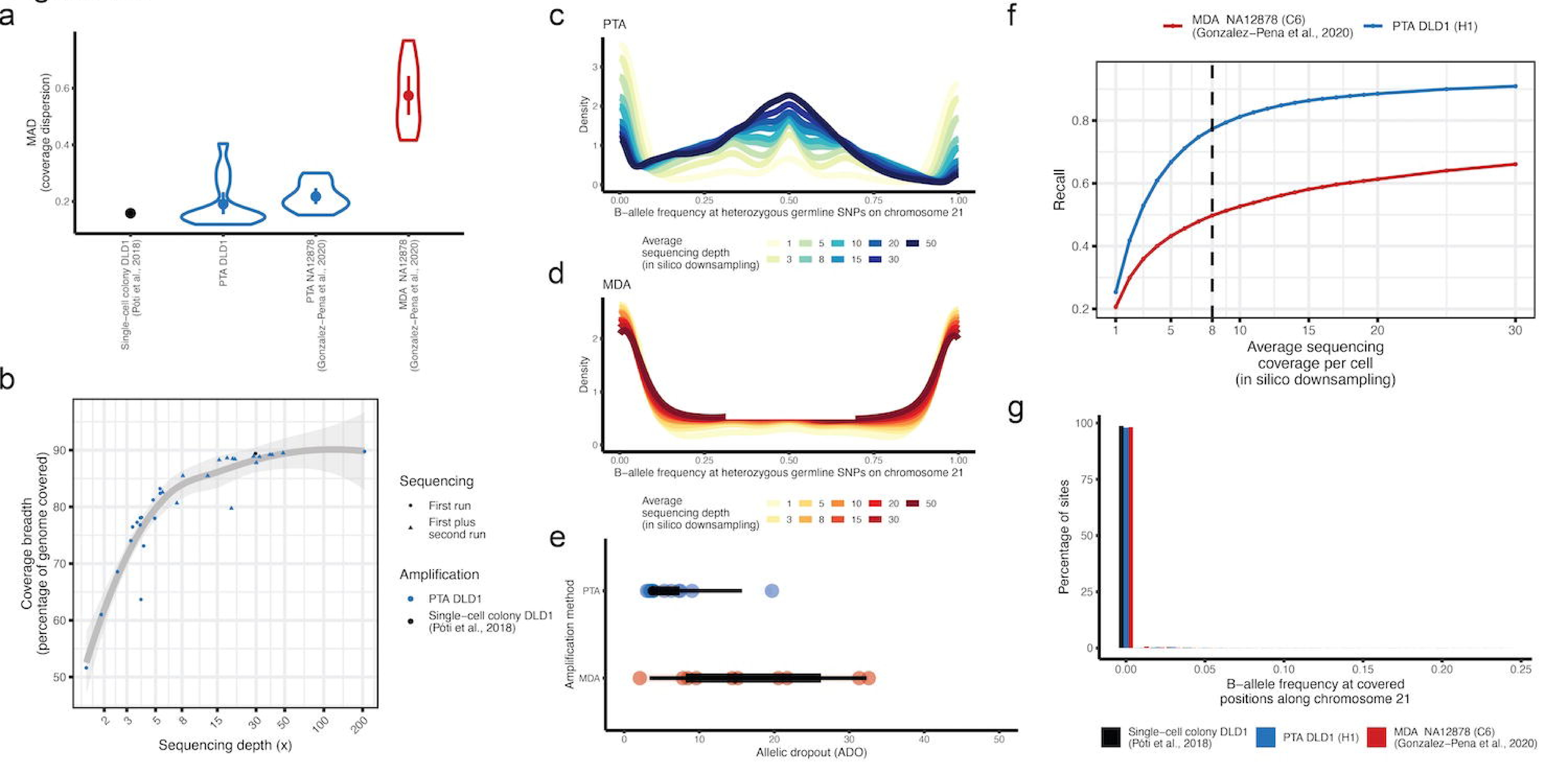

Figure S6

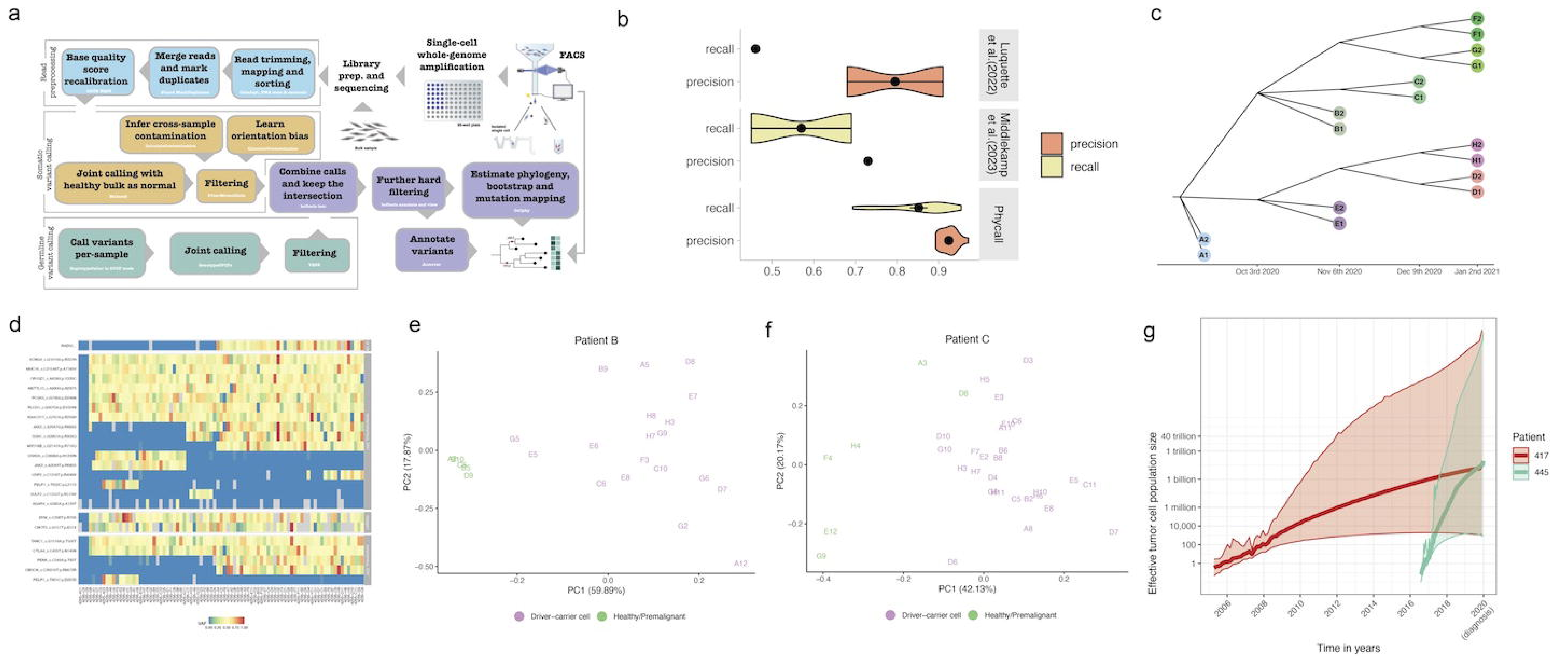
a
Read preprocessing
Base quality score recalibration
Merge reads and mark duplicates
Read trimming, mapping and sorting
Library prep. and sequencing
Single-cell whole-genome amplification
FACS
Somatic variant calling
Infer cross-sample contamination
Learn orientation bias
Joint calling with healthy bulk as normal
Filtering
Combine calls and keep the intersection
Further hard filtering
Estimate phylogeny, bootstrap and mutation mapping
Germline variant calling
Call variants per-sample
Joint calling
Filtering
Annotate variants
b
recall
precision
Luquette et al.(2022)
Middlekamp et al.(2023)
Phycall
precision
recall
0.5
0.6
0.7
0.8
0.9
c
Oct 3rd 2020
Nov 6th 2020
Dec 9th 2020
Jan 2nd 2021
d
e
Patient B
PC1 (59.89%)
PC2 (17.87%)
Driver-carrier cell
Healthy/Premalignant
f
Patient C
PC1 (42.13%)
PC2 (20.17%)
Driver-carrier cell
Healthy/Premalignant
g
Effective tumor cell population size
40 trillion
1 trillion
1 billion
1 million
10,000
100
1
Patient
417
445
2006
2008
2010
2012
2014
2016
2018
2020 (diagnosis)
Time in years
